# Integrated health service delivery during COVID-19: a scoping review of published evidence from low-income and lower-middle-income countries

**DOI:** 10.1136/bmjgh-2021-005667

**Published:** 2021-06-16

**Authors:** Md Zabir Hasan, Rachel Neill, Priyanka Das, Vasuki Venugopal, Dinesh Arora, David Bishai, Nishant Jain, Shivam Gupta

**Affiliations:** 1 School of Population and Public Health, The University of British Columbia, Vancouver, British Columbia, Canada; 2 Department of International Health, Johns Hopkins University Bloomberg School of Public Health, Baltimore, Maryland, USA; 3 Department of Health and Family Welfare, Government of Gujarat, Gandhinagar, India; 4 Department of Population, Family and Reproductive Health, Johns Hopkins University Bloomberg School of Public Health, Baltimore, Maryland, USA; 5 Deutsche Gesellschaft für Internationale Zusammenarbeit GmbH India Office, New Delhi, India

**Keywords:** review, COVID-19, health systems, public health, health services research

## Abstract

**Background:**

Integrated health service delivery (IHSD) is a promising approach to improve health system resilience. However, there is a lack of evidence specific to the low/lower-middle-income country (L-LMIC) health systems on how IHSD is used during disease outbreaks. This scoping review aimed to synthesise the emerging evidence on IHSD approaches adopted in L-LMIC during the COVID-19 pandemic and systematically collate their operational features.

**Methods:**

A systematic scoping review of peer-reviewed literature, published in English between 1 December 2019 and 12 June 2020, from seven electronic databases was conducted to explore the evidence of IHSD implemented in L-LMICs during the COVID-19 pandemic. Data were systematically charted, and key features of IHSD systems were presented according to the postulated research questions of the review.

**Results:**

The literature search retrieved 1487 published articles from which 18 articles met the inclusion criteria and included in this review. Service delivery, health workforce, medicine and technologies were the three most frequently integrated health system building blocks during the COVID-19 pandemic. While responding to COVID-19, the L-LMICs principally implemented the IHSD system via systematic horizontal integration, led by specific policy measures. The government’s stewardship, along with the decentralised decision-making capacity of local institutions and multisectoral collaboration, was the critical facilitator for IHSD. Simultaneously, fragmented service delivery structures, fragile supply chain, inadequate diagnostic capacity and insufficient workforce were key barriers towards integration.

**Conclusion:**

A wide array of context-specific IHSD approaches were operationalised in L-LMICs during the early phase of the COVID-19 pandemic. Emerging recommendations emphasise the importance of coordination and integration across building blocks and levels of the health system, supported by a responsive governance structure and stakeholder engagement strategies. Future reviews can revisit this emerging evidence base at subsequent phases of COVID-19 response and recovery in L-LMICs to understand how the approaches highlighted here evolve.

**Key questions**

**What is already known?**

- Integrated health service delivery (IHSD) is a promising approach towards Universal Health Coverage and can improve health systems resiliency during health emergencies.
- There is a lack of evidence on IHSD in low/lower-middle-income countries (L-LMICs), and there are no existing reviews on IHSD in L-LMICs during the COVID-19 pandemic.

**What are the new findings?**

- IHSD is occurring in L-LMICs during COVID-19, with the bulk of evidence coming from India.
- Horizontal and systematic integration was most reported in the literature, including the development of COVID-19 specific surveillance, testing, triage, quarantine and treatment protocols integrated into existing service delivery systems while maintaining routine health service delivery.
- A range of innovative approaches and integration typologies are also being operationalised, including the use of digital health technologies, integration with pharmaceutical and AYUSH (Ayurveda, Yoga and Naturopathy, Unani, Siddha and Homoeopathy—the six types of traditional or complementary medicine systems practiced in India) providers, triage algorithms for mental health referrals and leveraging military infrastructure.

**What do the new findings imply?**

- IHSD approaches are potentially viable for L-LMIC health systems during health emergencies; however, the design and operational approaches remain context-specific.
- Limited studies outside India were identified, which could either reflect more integration in the Indian health system, a higher COVID-19 burden in India than other L-LMICs at the time of the review, or increased publication opportunities from Indian authors.
- Additional research can update these emerging findings to explore how they evolve throughout the COVID-19 pandemic and to identify additional evidence from other contexts.

**Key questions**

**What is already known?**

- Integrated health service delivery (IHSD) is a promising approach towards Universal Health Coverage and can improve health systems resiliency during health emergencies.
- There is a lack of evidence on IHSD in low/lower-middle-income countries (L-LMICs), and there are no existing reviews on IHSD in L-LMICs during the COVID-19 pandemic.

**What are the new findings?**

- IHSD is occurring in L-LMICs during COVID-19, with the bulk of evidence coming from India.
- Horizontal and systematic integration was most reported in the literature, including the development of COVID-19 specific surveillance, testing, triage, quarantine and treatment protocols integrated into existing service delivery systems while maintaining routine health service delivery.
- A range of innovative approaches and integration typologies are also being operationalised, including the use of digital health technologies, integration with pharmaceutical and AYUSH (Ayurveda, Yoga and Naturopathy, Unani, Siddha and Homoeopathy—the six types of traditional or complementary medicine systems practiced in India) providers, triage algorithms for mental health referrals and leveraging military infrastructure.

**What do the new findings imply?**

- IHSD approaches are potentially viable for L-LMIC health systems during health emergencies; however, the design and operational approaches remain context-specific.
- Limited studies outside India were identified, which could either reflect more integration in the Indian health system, a higher COVID-19 burden in India than other L-LMICs at the time of the review, or increased publication opportunities from Indian authors.
- Additional research can update these emerging findings to explore how they evolve throughout the COVID-19 pandemic and to identify additional evidence from other contexts.

## Introduction

The COVID-19 has been one of the most significant healthcare emergencies in the past 100 years, claiming over 3.14 million lives worldwide from December 2019 to April 2021.^1^ Although initially concentrated in developed countries, the pandemic has increasingly taken a toll on low/lower-middle-income countries (L-LMICs),^2–4^ with India second in total COVID-19 cases.^1^ Health systems in L-LMICs have faced significant strain during the pandemic. Improving or expanding case surveillance, contact tracing, communications campaigns, combating misinformation and maintaining access to essential health services were established as the risk mitigation strategies.^5–7^ However, the fragmented nature of health service delivery in L-LMICs poses extraordinary challenges to meet the dual goal of pandemic response and routine service continuity.^8^

### Integrated health service delivery during COVID-19 pandemic

Integrated, people-centred health systems are increasingly considered a central component of Universal Health Coverage and are globally recognised with an adopted resolution of the 69th World Health Assembly in 2016.^9^ ‘Integration’ of the health service delivery has many meanings in global health policy and systems research. However, an all-encompassing and appropriate definition provided by the WHO Regional Office for Europe characterised the integrated health service delivery (IHSD) system as:

An approach to strengthen people-centered health systems through the promotion of the comprehensive delivery of quality services across the life-course, designed according to the multidimensional needs of the population and the individual and delivered by a coordinated multidisciplinary team of providers working across settings and levels of care … with feedback loops to continuously improve performance and to tackle upstream causes of ill health and to promote well-being through intersectoral and multisectoral actions.^10^

Integrated care systems are characterised into four typologies,^11^ which includes: (a) organisational integration, where different organisations coordinated with each other using a single governing structure, (b) functional integration, when non-clinical services were integrated to facilitate health service delivery, (c) service integration, where multiple providers and/or facilities across the level of health system organise themselves for service provisions, and (d) clinical integration, when providers or facilities streamlines their clinical care procedures based on a standardised protocol for care.

However, these four typologies are not mutually exclusive. One or any combinations of the typologies may be present while implementing an IHSD model across the primary, secondary or tertiary level of care—also known as vertical integration^12^—or integrating multiple operating units and/or organisations at the same stage of the health system, known as horizontal integration.^13^ Regardless of the integration structure—vertical, horizontal or a mix of both—the IHSD system can be integrated via two mutually exclusive mechanisms.^10^ When the integration was based on the ethos of shared understanding, mutual collaboration and trust, it is defined as normative integration. On the other hand, systematic integration is led by specific policies and guidelines adopted across the organisational and health system levels.

Integrated service delivery is increasingly being emphasised as countries focus on improving the overall resiliency of their health systems.^14–16^ However, the goals of IHSD reforms and the modalities of implementation often differ across high-income, middle-income, lower-middle-income and low-income country’s health systems. In L-LMICs, most IHSD approaches aim to increase access, coverage and efficacy of specific services for predefined populations,^17^ including integrating vertical services in primary care^18^ or merging of multiple vertical services into a common delivery package or intervention. The integration processes are often observed at the facility or service delivery level, particularly for HIV/AIDS, tuberculosis, family health and reproductive health services.^17–19^ However, the evidence base for IHSD is still nascent^17–20^ and often focused on over-simplified debates of vertical versus horizontal service delivery structure.^21^

While exploring the history of previous disease outbreaks, it is very much evident that an IHSD model is well suited in response to all four phases of a pandemic^14^—(a) interpandemic: the period between the pandemics, (b) alert: when a new disease with pandemic potential has been identified in humans, (c) pandemic: period of the global spread of the disease and (d) transition: de-escalation of response and movement towards recovery as risk is reduced across the world. The potential benefit of the integrated care approach is well documented during the HIV/AIDS pandemic in sub-Saharan Africa^22^ and pandemic influenza in the USA.^23^ Since the emergence of COVID-19, new evidence is emerging—mainly from the developed countries, such as the UK,^24^ Italy,^25^ Greece and Spain^26^—which has demonstrated a promising outcome of the IHSD approach.

However, there is a dearth of evidence from the L-LMICs on the effect of the IHSD system when COVID-19 is overwhelming their strained resources and fragmented healthcare system.^27^ According to the Global Health Security index, developed in 2019,^28^ most L-LMICs are least-prepared in response planning and operationalising health services during a potential pandemic. Considering the fragmented health systems and limited capacity of L-LMICs, they are highly likely to encounter considerable challenges in effective and timely response to COVID-19. However, in countries like Bangladesh, India and Vietnam, the government response to the pandemic was as stringent as some developed countries.^29^

For instance, according to the Oxford COVID-19 Government Response Tracker, the stringency of Vietnam and the USA are at the same level (stringency index=56.94). While we cannot directly compare the strategic response of these two countries against COVID-19, Vietnam’s experience with containment of the SARS epidemic may have provided them valuable lessons in pandemic response.^30^ Following their experience in managing SARS, Vietnam designed to mobilise an integrated and comprehensive response with the community and preventive healthcare services, acting together as one united workforce.

Innovation in the IHSD system that emerged from a limited resource setting can provide critical insight for rapid response and decisive action to manage the ongoing or future pandemics. This scoping review aims to compile the existing published evidence of the integrated service delivery approach adopted in response to the COVID-19 pandemic in the L-LMICs, systematically map the features, and build the knowledge base of the IHSD systems for practical and evidence-based decision making.

## Methods

We have followed the scoping review framework developed by Arksey and O’Malley to structure and implement this scoping review,^31^ adhering to the checklist of PRISMA Extension for Scoping Reviews^32^ (see online supplemental file 1 for more details). The collection, screening, synthesis and reporting of evidence in this scoping review adhered to the following five steps: (a) conceptualising the research questions, (b) identification of relevant peer-reviewed literature, (c) selection of the studies from electronic databases, (d) charting of evidence and (e) collation and synthesis of the data. The detailed protocol of this review is registered at the OSF,^27^ and we encouraged our readers to review the published protocol of this review.^27^

10.1136/bmjgh-2021-005667.supp1 Supplementary data

### Conceptualising the research questions

In this scoping review, we have aimed to explore published evidence on the IHSD systems implemented in the L-LMICs in response to the COVID-19 pandemic. To achieve this aim, we have tried to answer the following research questions:

1. What are the features of the IHSD systems in the L-LMICs during the COVID-19 pandemic?
2. How were the IHSD systems operationalised within the health systems of L-LMICs to provide healthcare in the context of the COVID-19 pandemic?
3. Considering the opportunities and challenges posed while implementing the IHSD system in L-LMICs, what recommendations can be made for COVID-19 preparedness, response and recovery?

While answering these research questions, we used the broad definition of IHSD proposed by WHO,^10^ and considered service integration during COVID-19 as—(a) integration of newly developed COVID-19 response activities within the existing health system; (b) integration of specific aspects of the existing health service provision within the COVID-19 response that had relevance for the overall health system and (c) integration of services to support continuity of routine health systems operations during the COVID-19 pandemic.

### Identification of relevant peer-reviewed literature

To identify the initial pool of peer-reviewed literature on COVID-19, a comprehensive search strategy was implemented with a wide range of keywords and search terms related to four primary concepts: (a) ‘integrated health service delivery’, (b) ‘COVID-19’, (c) ‘pandemic preparedness’ and (d) ‘low and lower-middle income countries’. We conducted a systematic search of the literature in seven electronic databases: PubMed/MEDLINE, Scopus, EMBASE, Web of Science, CINHAL Plus, LitCovid and the WHO COVID-19 literature database. We have restricted the search parameters within an article published in the English language, considering the feasibility of the study. The complete search strategy for PubMed/MEDLINE is provided in online supplemental file 2.

10.1136/bmjgh-2021-005667.supp2 Supplementary data

### Study selection

The search was implemented across the seven electronic databases on 12 June 2020. Title, abstract and the citation of the searched articles were imported into the Covidence systematic review software (covidence.org) system, which facilitated the removal of duplicates and screen the articles for eligibility. The screening was conducted in two stages—(a) review of title and abstracts and (b) screening of full text—based on predefined eligibility conditions presented in table 1. To align these criteria with our specific research questions, we have considered the ‘Population-Concept-Context’ framework^33^ to develop the inclusion and exclusion criteria.

**Table 1:** Inclusion and exclusion criteria for the study selection process of the scoping review

	Inclusion criteria	Exclusion criteria
Concept	Integrated health service delivery system	Article without evidence or discussion on integrated health service delivery (eg, a case report on patients with COVID-19 which recommend implementation of integrated health service delivery, and it did not explore any such systems)
Context	Health service organised during COVID-19 pandemic	
Population	Low-income countries and lower-middle-income countries	Countries from the upper-middle-income and high-income categories
Article type	Original research, case studies or case reports, commentary or editorial, systematic, scoping, or rapid review, research letter	Author’s reply or opinion, research highlight, news or media watch
Time frame	1 December 2019–12 June 2020	
Reporting	Published peer-reviewed articles Articles written in the English language	Article not published in English or without translation

Low-income economies are defined as Gross National Income (GNI) per capita of $1035 or less in 2019 (n=29). Lower-middle-income economies are defined as GNI per capita $1036 and $4045 (n=50) (https://datahelpdesk.worldbank.org/knowledgebase/articles/906519-world-bank-country-and-lending-groups, accessed 26 April 2020).

Studies that did not explore any implementation of the IHSD system in response to the COVID-19 pandemic in L-LMICs were excluded during the screening process. We included a wide range of literature, such as original articles, protocols, editorials and commentaries, published in the English language between 1 December 2019 and 12 June 2020; however, news and media watch, author’s reply and research highlights were excluded, as they often do not offer the full context of the evidence. Three researchers independently conducted the screening process, with any undisputed disagreement for an article’s inclusion that was adjudicated on by a senior researcher.

### Charting of evidence

Next, all eligible articles were re-read, and evidence on IHSD was charted using a standardised data extraction template in Microsoft Excel. As a test extraction exercise, three researchers charted data from five articles, and the result was triangulated to develop a shared understanding. After completing the data extraction, the entire team reviewed the results to ensure the consistency and robustness of the analysis. Details of the data elements extracted during the charting process are provided in online supplemental file 2.

### Collating, synthesising, and reporting the results

First, we summarised the place of origin, objective and design of the studies. The evidence of IHSD systems during the COVID-19 pandemic was summarised into thematic areas to answer the postulated research questions of this scoping review. We have organised the characteristics of IHSD systems according to their implementation during different phases of a pandemic (such as alert, pandemic, transition and interpandemic),^14^ their structure, and mechanism as a part of the IHSD system. We have also explored the example of integration across all health systems building blocks, informed by recent work by Salam *et al*,^34^ which used the nomenclature of the building blocks to compare integration across components of the health system. Finally, the integrated system’s features were described based on the typology of the integration—clinical, service, functional and organisation. Using a narrative format and with the help of tables, we have reported the result of this scoping review in the next section.

### Patient and public involvement

This review was conducted using previously published peer-reviewed literature. Thus, no patients or the public were involved in the planning, design, data acquisition, analysis and dissemination of the study result.

## Results

### Selection and features of the evidence on IHSD system

**Figure 1:**
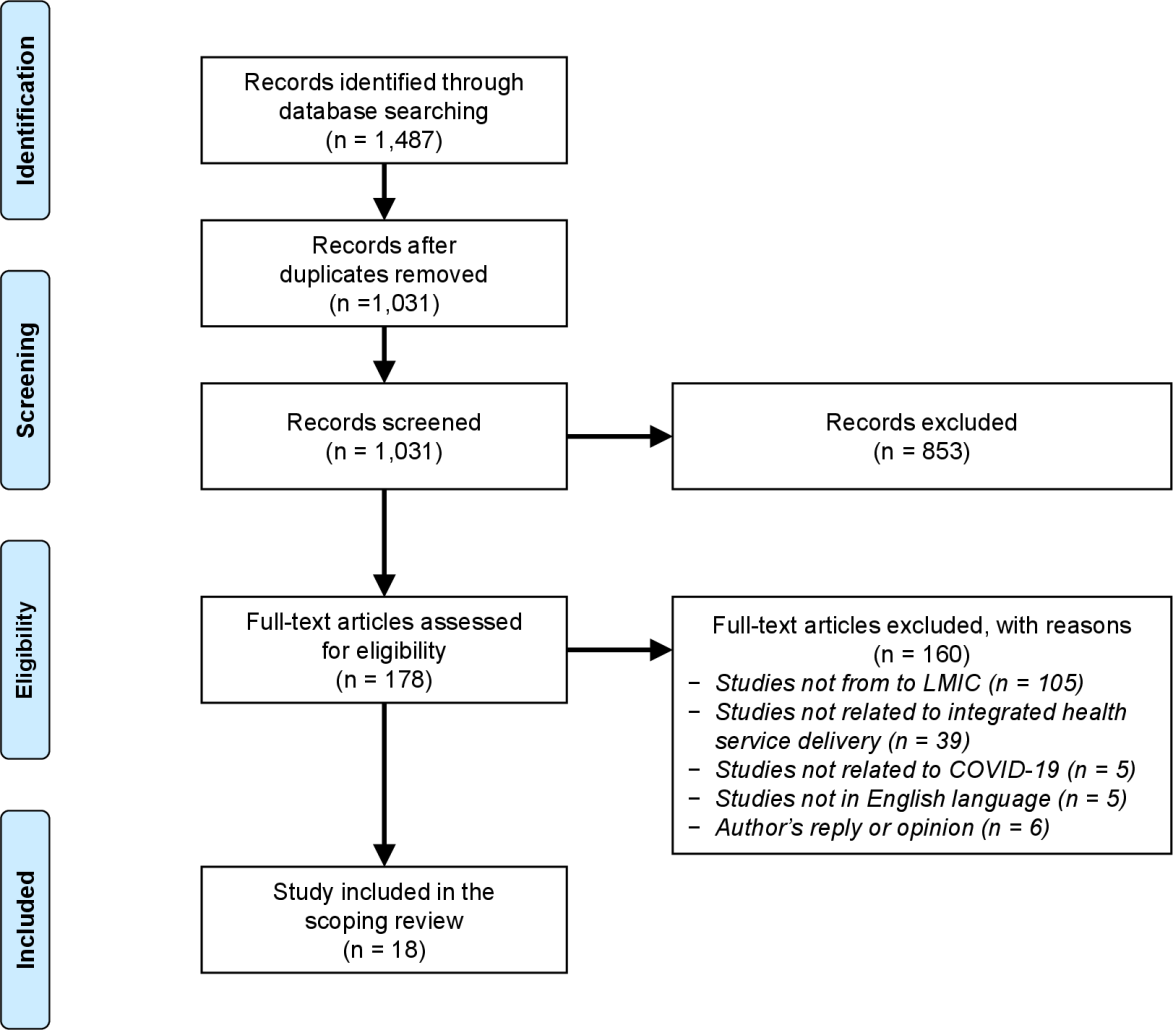
Preferred Reporting Items for Systematic Reviews and Meta-Analyses flow chart. LMIC, lower-middle-income country.

The search process retrieved 1487 published articles from the seven databases. From the pool of retrieved articles, 456 duplicates were removed, and 1031 articles were selected for screening. In total, 853 studies were excluded during the title and abstract review process, and additional 160 articles were excluded after full-text review. In total, 18 articles were included in the scoping review after full-text review. The result of the searching, screening and study selection process is summarised in figure 1 according to the PRISMA chart.^35^

The majority of the articles included in the review originated from the WHO South-East Asia region (n=14), including 12 studies from India and 1 study from Nepal and Vietnam. The remaining studies are from Tunisia, Bolivia, African Region (information reported from Algeria, Cameroon, Cote d’Ivoire, Gambia, Madagascar, Nigeria, Rwanda, Senegal, South Sudan, Uganda) and East Mediterranean Region (information reported from Egypt, Iraq, Jordan, Morocco, Saudi Arabia, Sudan, Tunisia). While most of the articles were commentary or editorial (n=7) and reviews (n=6), the eligible articles also included three observational studies and two intervention protocols.

### Operational features of the IHSD system with the health systems of L-LMICs

Table 2 presents the operationalisation of IHSD systems reported within the selected studies considering the context of COVID-19 and based on their primary focus on the phase of the pandemic, the structure and mechanism of integration and the health systems building blocks considered to be integrated as part of the IHSD effort.

**Table 2:** Operational features of the integrated health service delivery system identified from the 18 studies included in the scoping review

Included articles	Country or geography	Primary focus on the pandemic continuum				Health systems building blocks involved in the integration						Structure of integration			Integration mechanism	
		Interpandemic phase	Alert phase	Pandemic phase	Transition phase	Service delivery	Health workforce	Medicine & technology	Health information system	Health financing	Governance	Vertical	Horizontal	Both	Systematic	Normative
Al Nsour *et al*^40^	Eastern Mediterranean Region*		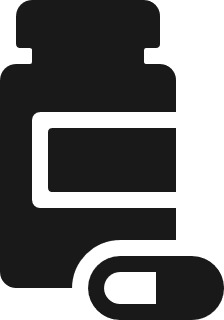			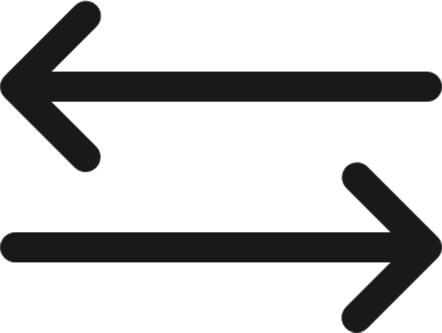	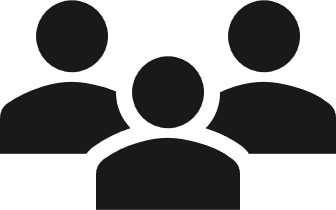				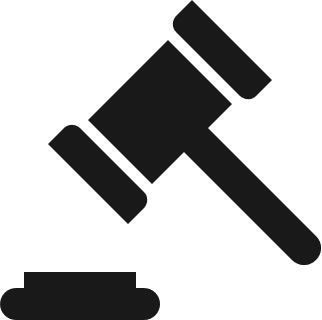			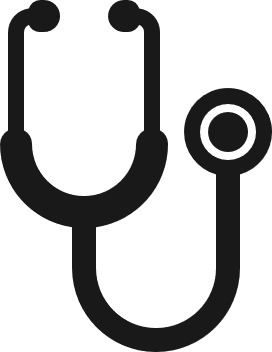	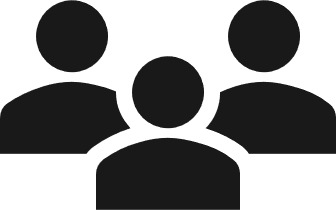	
Banerji^41^	India		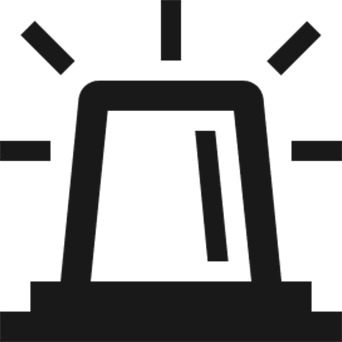			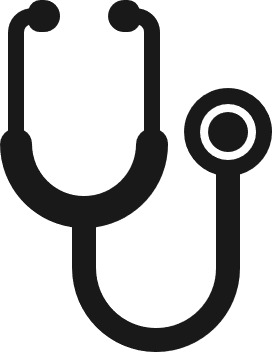	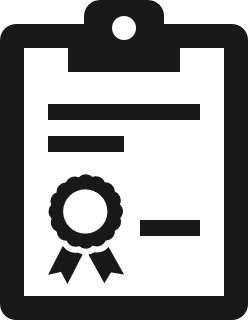	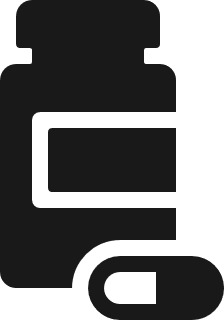			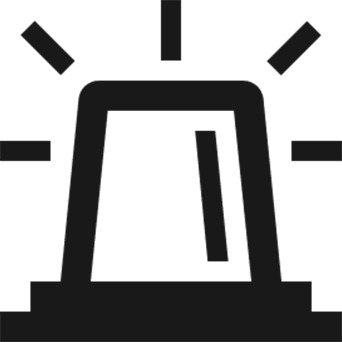			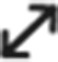	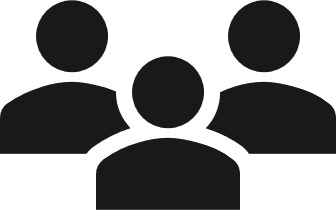	
Chellamuthu and Muthu^45^	India			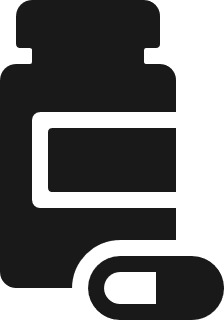		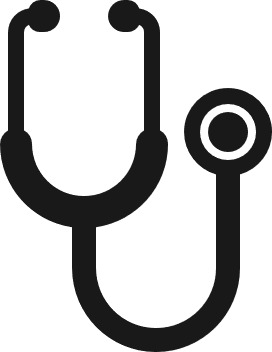	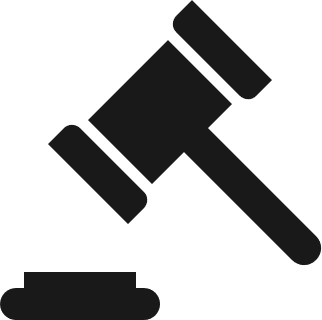						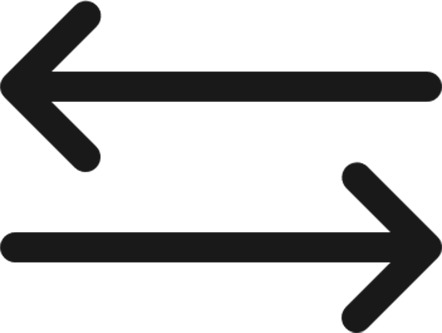		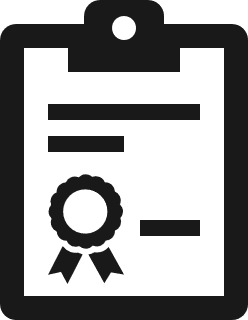	
Garg *et al*^46^	India			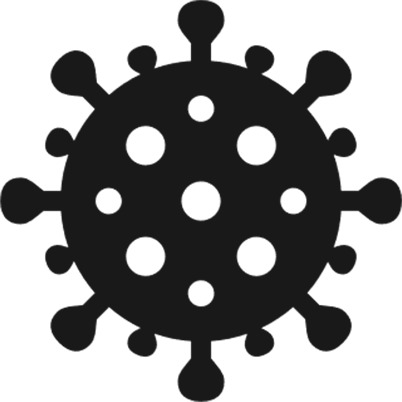			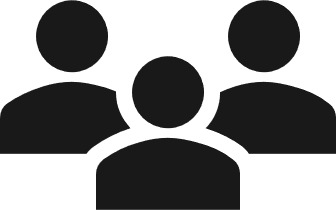	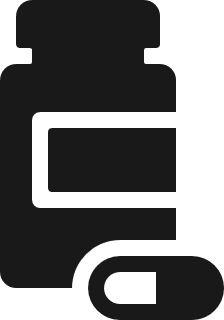					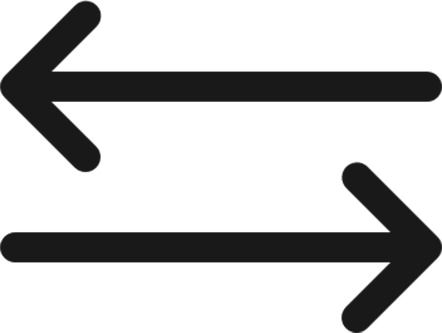			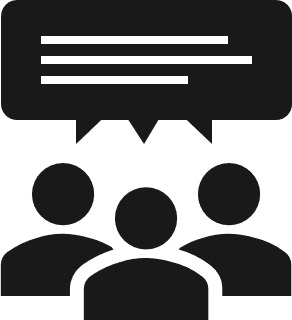
Gupta *et al*^42^	India		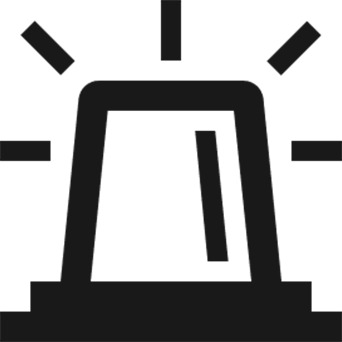			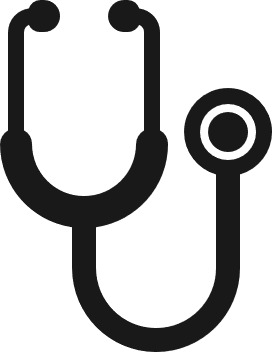	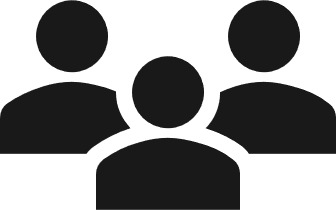	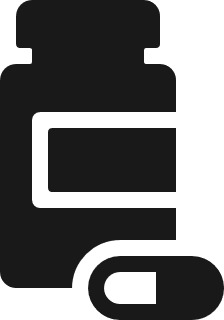	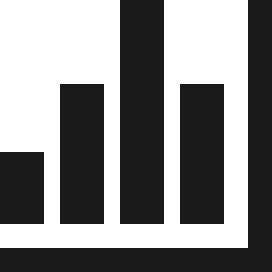		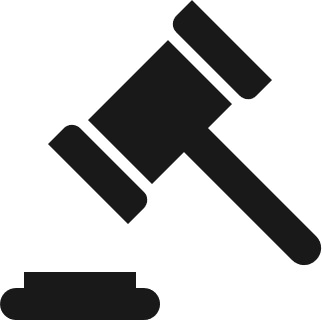			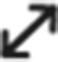	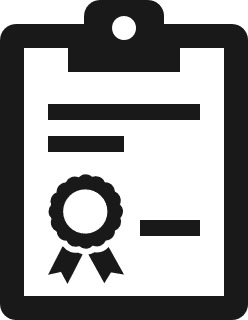	
Gupta *et al*^43^	India		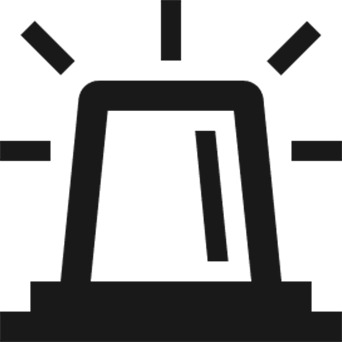			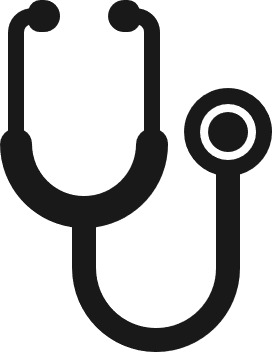		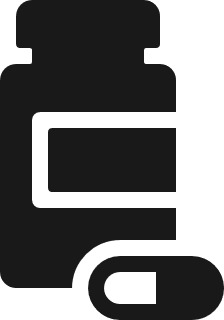	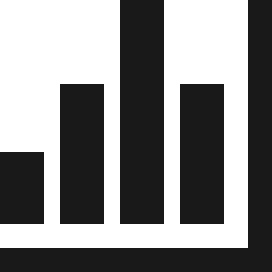		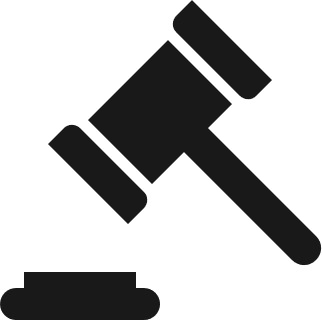			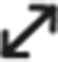	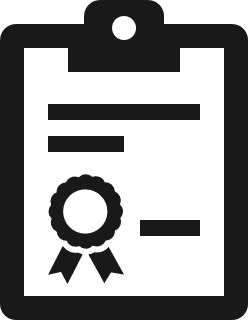	
Ha *et al*^37^	Vietnam		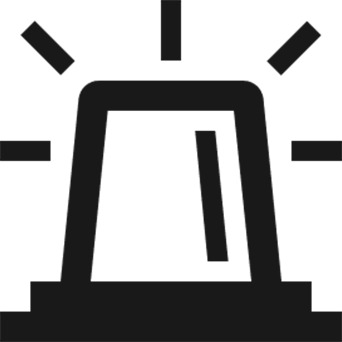	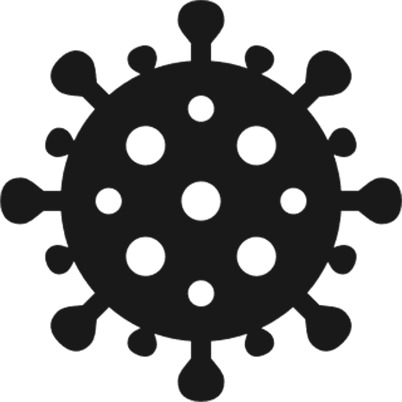		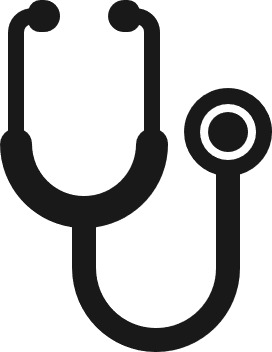	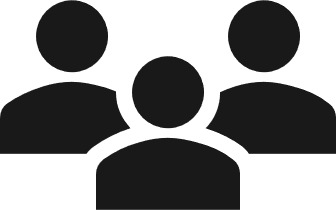	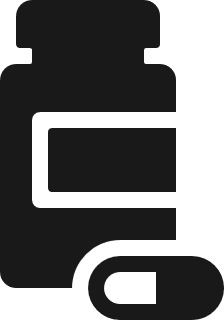			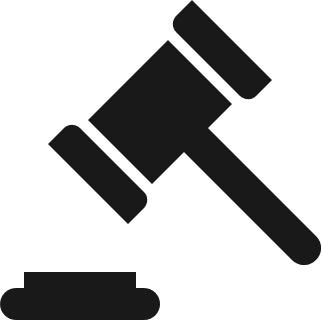	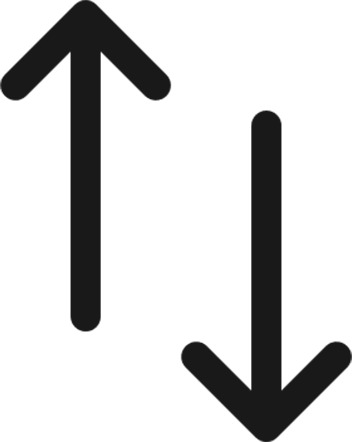			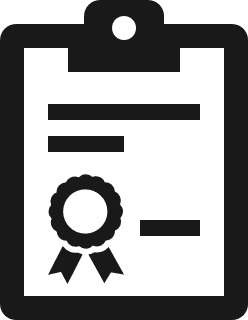	
Iyengar *et al*^47^	India			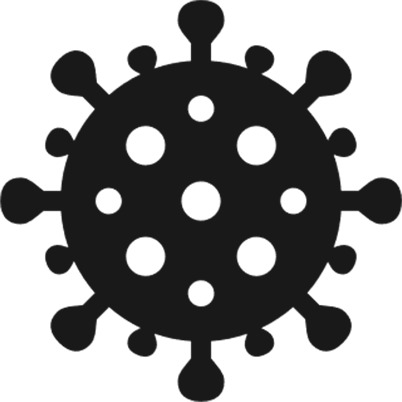					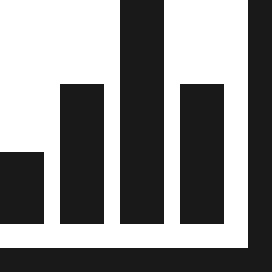				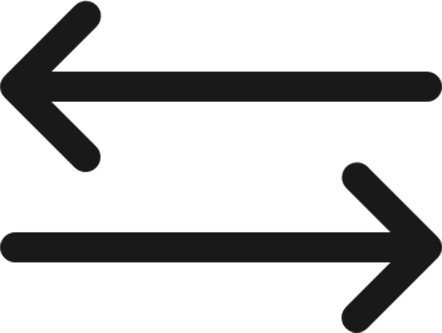		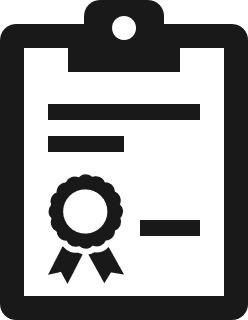	
Kaplan *et al*^38^	Bolivia		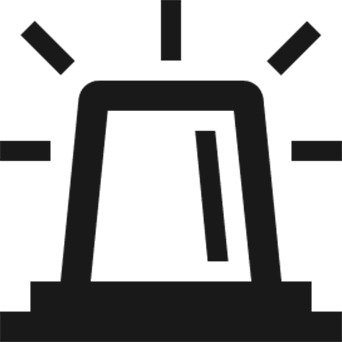	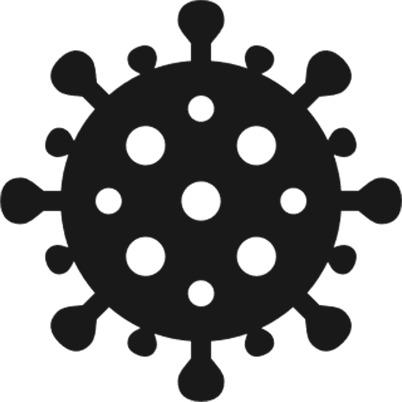		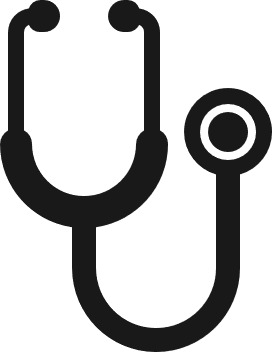		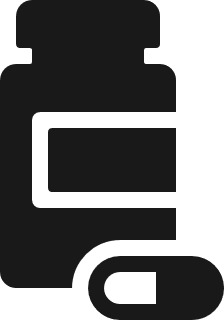			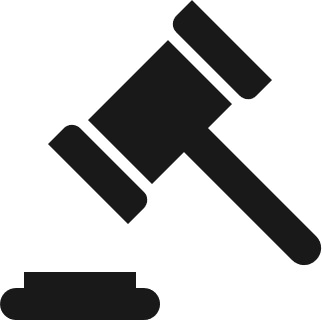			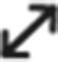	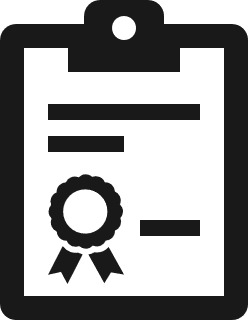	
Lal *et al*^50^	India			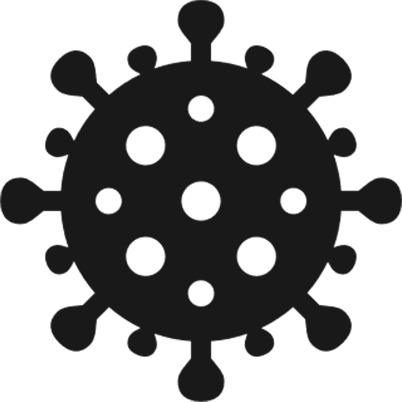		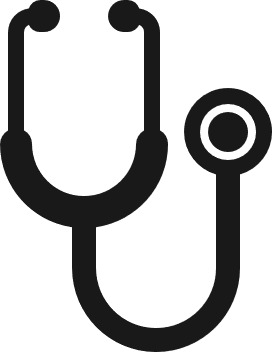	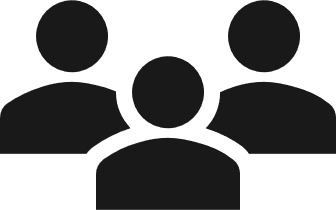	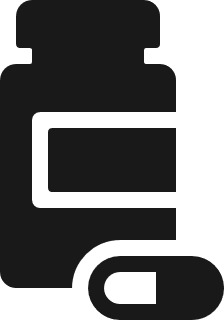	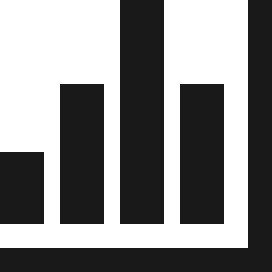				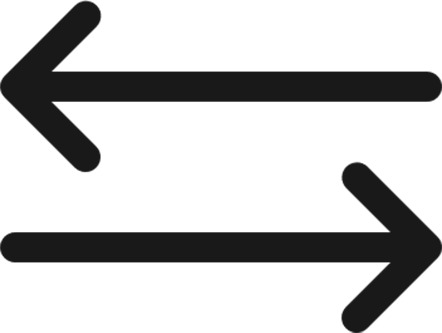		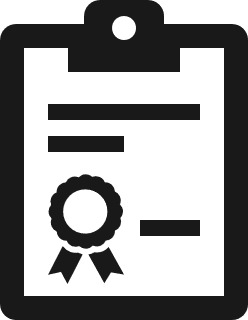	
Lucero-Prisno *et al*^39^	African region†		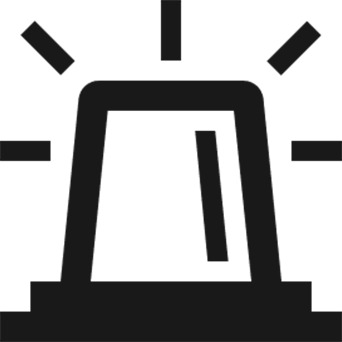	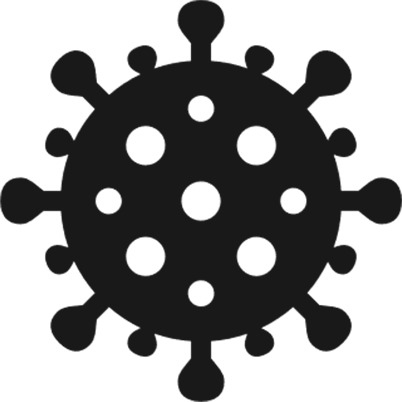		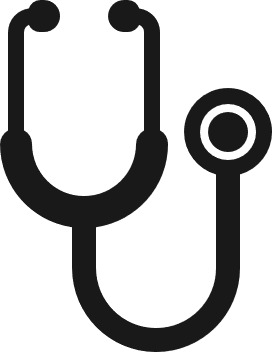	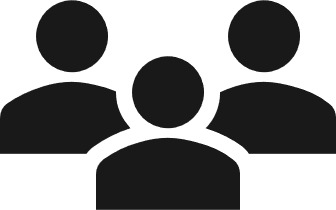	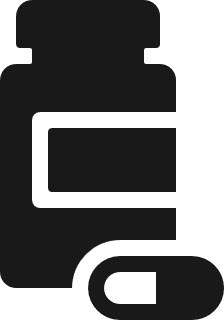	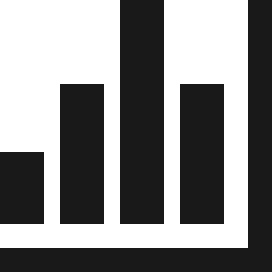		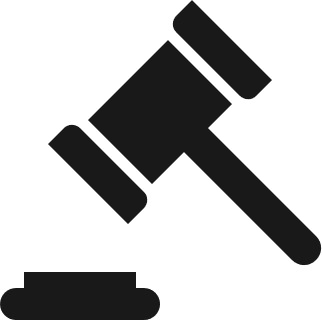			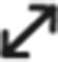	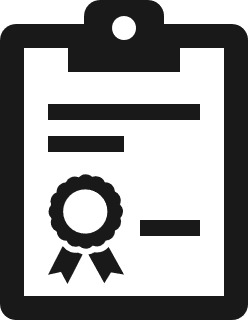	
Meghana *et al*^51^	India			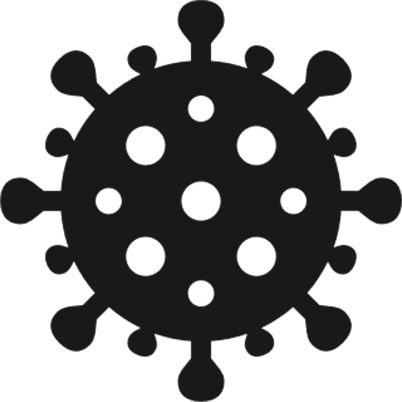		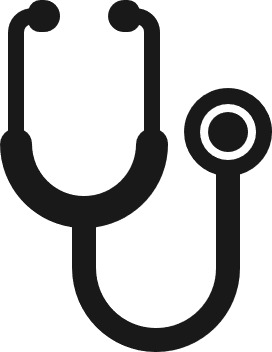	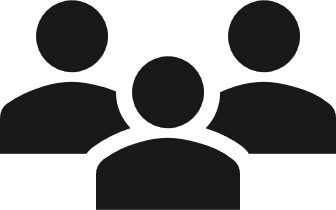	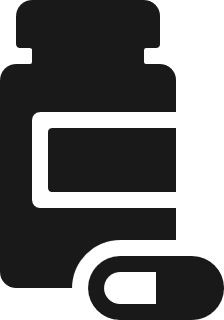					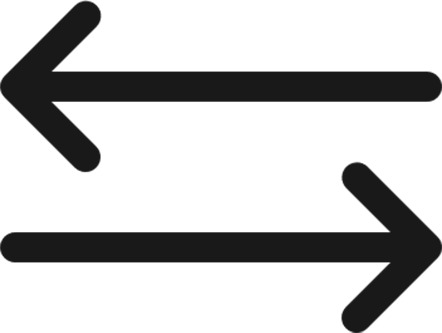			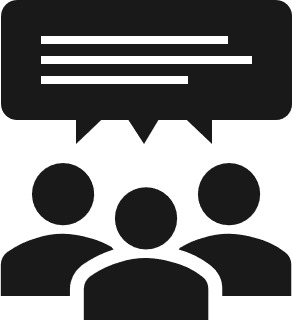
Meghwal *et al*^52^	India			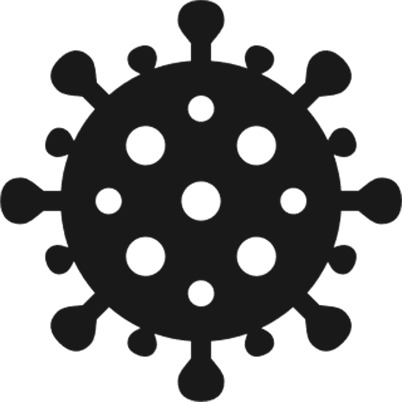		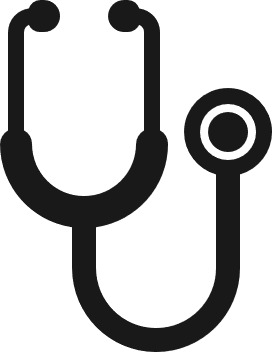	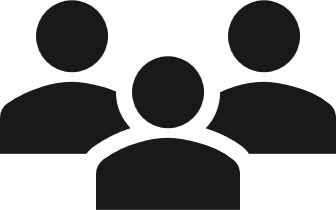	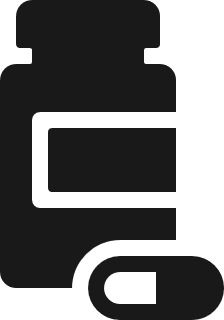	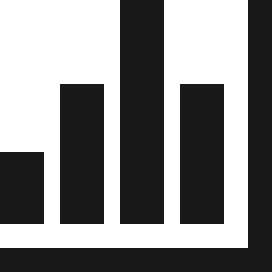		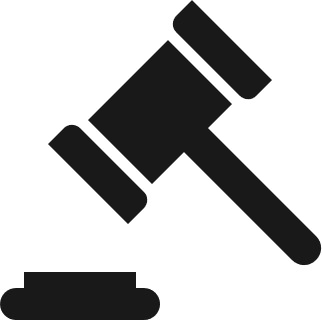		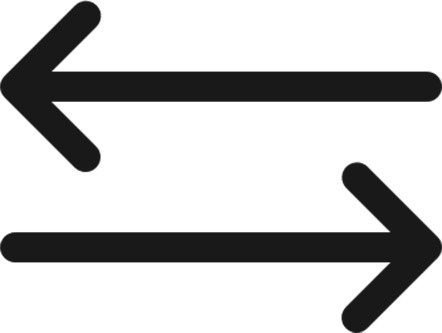		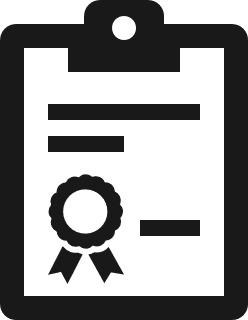	
Piryani *et al*^44^	Nepal		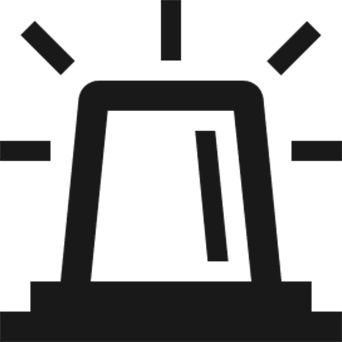			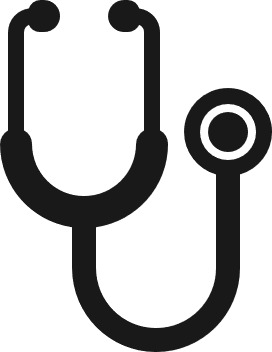		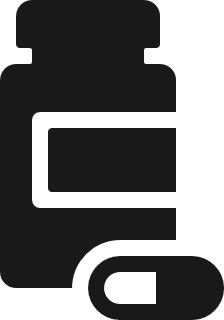			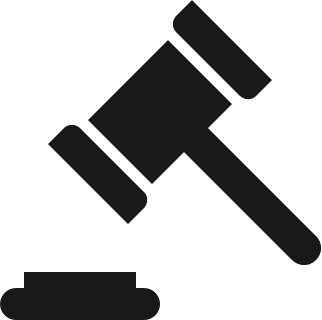			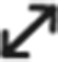	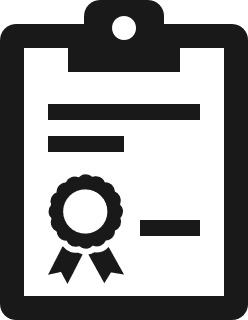	
Rastogi *et al*^48^	India			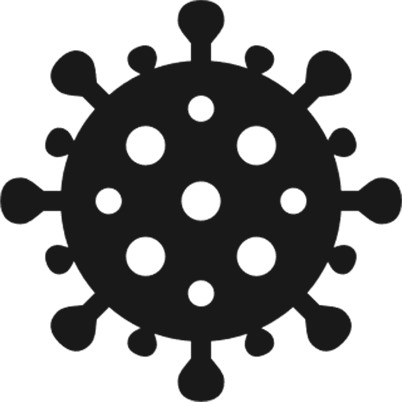		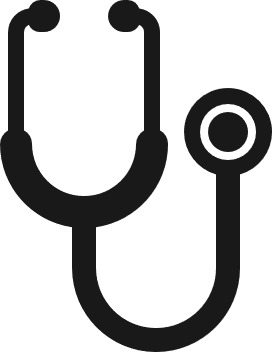		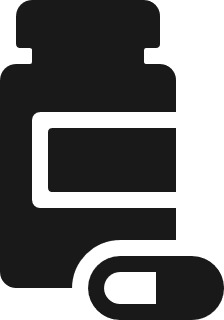					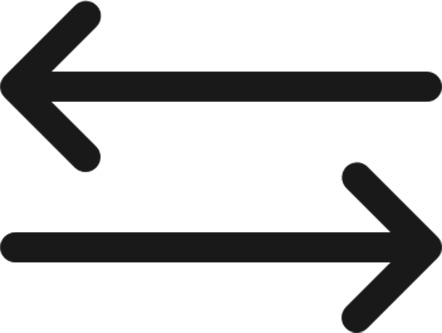			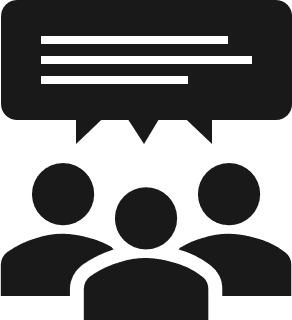
Rastogi *et al*^49^	India			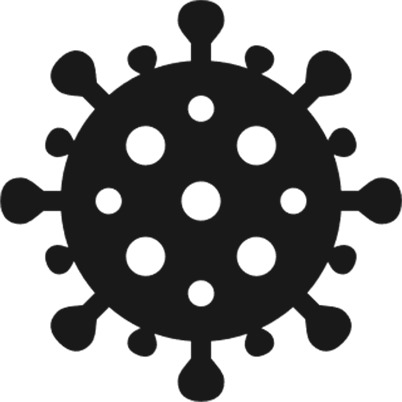		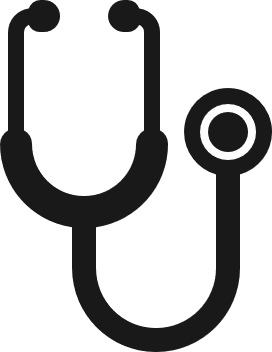		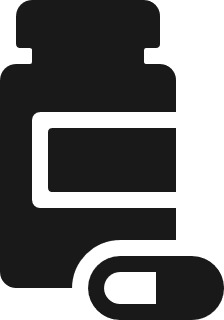					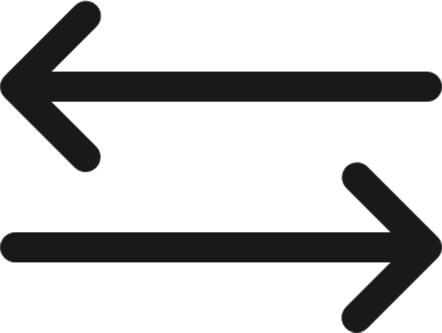			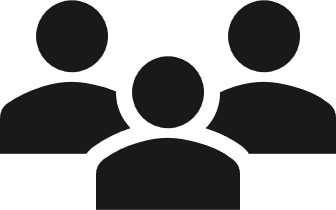
Shinde *et al*^53^	India			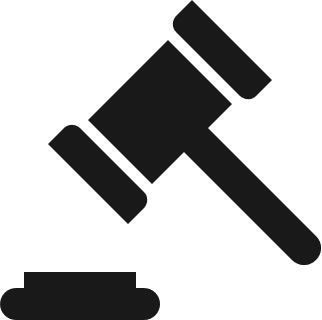		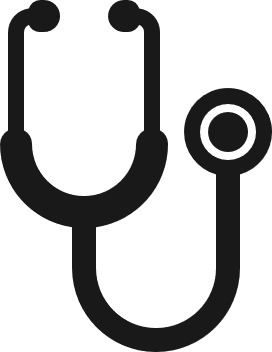	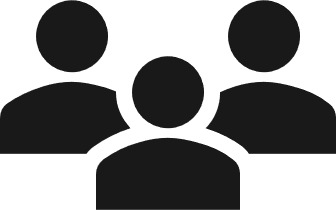	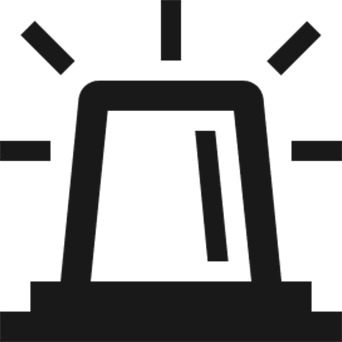					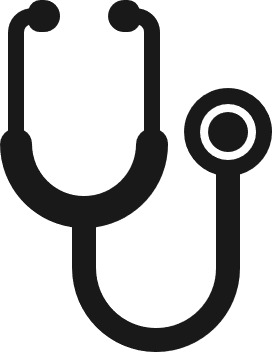		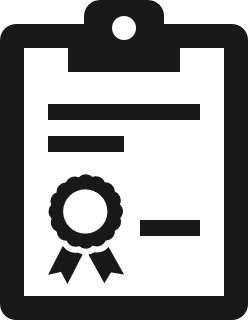	
Zgueb *et al*^36^	Tunisia	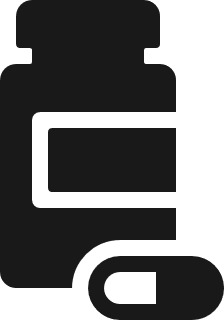	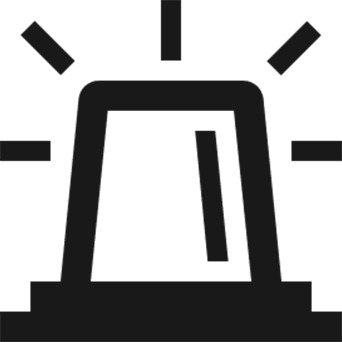			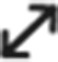	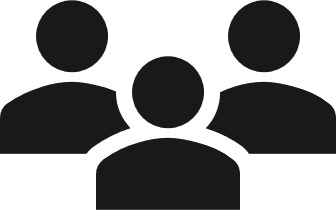	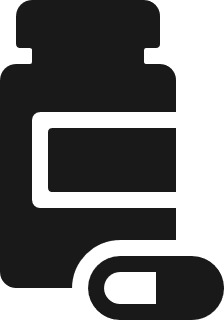	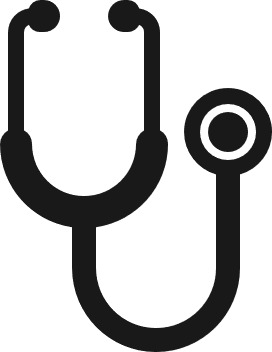		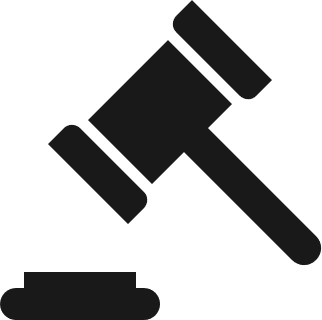		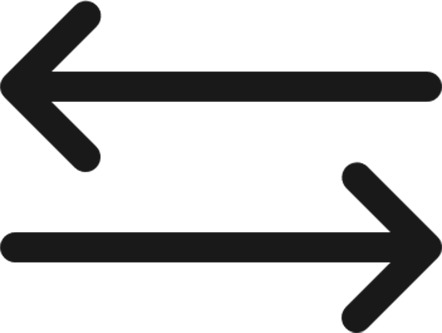		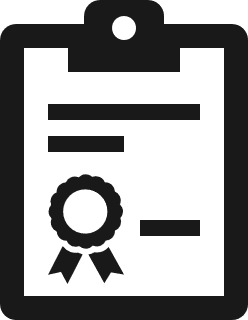	

18 studies were included in the scoping review, which met the inclusion criteria.

*Egypt, Iraq, Jordan, Morocco, Saudi Arabia, Sudan, Tunisia.

†Algeria, Cameroon, Cote d’Ivoire, Gambia, Madagascar, Nigeria, Rwanda, Senegal, South Sudan, Uganda.

The majority of the study focused on either alert or the pandemic phase while implementing the IHSD system, except Zgueb *et al*,^36^ which focused both on the interpandemic and alert phases to describe the development and implementation of a novel psychological crisis response intervention in Tunisia. Nonetheless, the article alluded to the necessity of building a well-trained health workforce system that goes above and beyond the timespan of the current pandemic. Three of the remaining 17 studies focused on both the alert and pandemic phase^37–39^ and no study included information related to the transition phase. All 18 studies included in this review described IHSD systems that integrated multiple health system building blocks. However, it was interesting to observe that IHSD systems implemented during the ‘alert phase’^40–44^ generally integrated a higher number of health system building blocks, compared with the IHSD system exclusively focused on the ‘pandemic phase’,^45–49^ except for Lal *et al*,^50^ Meghana *et al*,^51^ Meghwal *et al*^52^ and Shinde *et al.*^53^

According to our findings, service delivery, health workforce and medicine and technologies are the three most frequently integrated health system building blocks. Out of 18 studies, 7 reported integration of health information systems,^36 39 42 43 47 50 52^ and 10 reported integration of governance structure with other building blocks in response to COVID-19.^36–44 52^ While contrasting the pandemic continuum with the health systems building blocks (table 1)—no study exclusively focused on the pandemic phase—incorporated governance with the IHSD system, except Meghwal *et al.*^52^ Meghwal *et al*^52^ reported formalisation of a Rapid Response Team (RRT) to contain a COVID-19 cluster in a health facility in Rajasthan, India, with the help of a multidisciplinary group of experts from medical colleges, District Epidemiologist of Integrated Disease Surveillance Programme, and Surveillance Medical Officer of National Polio Surveillance Programme WHO India. None of the studies included in this review reported integrating healthcare financing structure (revenue generation, pooling or purchasing strategies) while responding to the COVID-19.

Almost 55% (n=10) of the studies implemented IHSD via a horizontal structure of integration.^36 45–53^ This variant of integration structure incorporates health services and health systems components within a single level of the health system or with a healthcare facility. The second most common integration structure—reported in seven studies^38–44^—was a mix of horizontal and vertical integration, where a multipronged approach was taken to execute a system-wide response against COVID-19. The only example of vertical integration was identified in Ha *et al*,^37^ highlighting specific measures adopted across the primary and secondary care systems. Finally, most of the studies (n=14) systematically implemented the IHSD models with guidelines and protocols specifically developed for the COVID-19 pandemic. Only four studies reported more of a normative mechanism of IHSD implementation,^46 48 49 51^ where no COVID-19 specific guideline was implemented; instead, the existing health systems structures and guidelines were adopted in response to the pandemic. Interestingly, all four of these studies were associated with implementing the IHSD system at the pandemic phase (table 2).

Regardless of the structure or mechanism of IHSD described in the studies, 72% (n=13) studies reported implementing multiple typologies of integration simultaneously. Among the 18 studies included in the scoping review, 7 studies described the IHSD system, which contains all four integration typologies (clinical, service, functional and organisational),^36 37 39 41–44^ 5 studies reported implementing three typologies of integration,^38 40 45 50 52^ 1 reported a combination of two typologies^51^ and 5 studies reported only one typology of integration.^46–49 53^ Considering the individual typology of health system integration, the functional variant was most frequently applied—either independently^46^ or in combination with other typologies.^36–47 50–52^ This was followed by service integration in 14 studies,^36–45 49–52^ clinical integration in 11 studies^36 37 39 41–45 48 50 53^ and finally organisational integration was observed in 10 studies.^36–44 52^ Table 3 presents the objective, designs and typologies of the 18 included articles with a detailed description of their IHSD design.

**Table 3:** Summary integrated health service delivery system of the 18 studies included in the scoping review (ordered alphabetically according to the name of the first author)

Study	Country or geography	Study objective	Study design	Typology and features of integrated health service delivery
Al Nsour *et al*^40^	Eastern Mediterranean region (EMR) (Egypt, Iraq, Jordan, Morocco, Saudi Arabia, Sudan, Tunisia)	This article elaborates on the response of the Global Health Development and Eastern Mediterranean Public Health Network, and the Field Epidemiology Training Programmes (FETPs) during the COVID-19 pandemic	Commentary or editorial	**Service:** Screening and surveillance activities conducted by FETPs at the point of entries in the EMR countries. **Functional:** Collation, synthesis and dissemination of information by the Public Health Emergency Management Centre in response to the pandemic. Conducting orientation session with physicians and public health practitioners to build a shared understanding of the protocols, case definitions and public messaging strategies. **Organisational:** Collaborating with FETPs by providing technical supports and educational materials.
Banerj^41^	India	This article summarises the Indian Armed Forces Medical Services (AFMS) response to the COVID-19 pandemic	Commentary or editorial	**Clinical:** With the guidance of the Ministry of Health and Family Welfare, AFMS developed a standard operating procedure (SOP) to establish quarantine facilities. **Service:** Armed force medical facility was designated as a COVID-19 treatment hospital with COVID-19 testing facilities. Service delivery was separated for patients with COVID-19 and non-COVID-19 for both inpatient and outpatient facilities. **Functional:** Aircraft from Indian Air Force were used to establish a supply chain of personal protective equipment (PPE), clinical equipment and medication. General duty soldiers were recruited as volunteers and underwent training for COVID-19 pandemic response, and participated in the implementation of preventive measures. **Organisational:** AFMS formalised rapid response medical teams and coordinated with local, state and federal government in screening, isolation and COVID-19 case management in the quarantine facilities.
Chellamuthu and Muthu^45^	India	This article explores the management of orthopaedic care in a tertiary care hospital using a pandemic response protocol during the COVID-19 pandemic	Review	**Clinical:** Maintaining inpatient visitor record, perform screening and record the full history by strictly following infection control measures. **Service:** Creating a separate group of physicians to provide inpatient and outpatient service without engaging with each other. Reducing elective surgical care. Using telemedicine and online tools to provide rehabilitative and postoperative care. **Functional:** Including physicians in the pandemic response task force, providing appropriate training to the physicians in pandemic response.
Garg *et al*^46^	India	This article highlights the preparedness of 51 primary healthcare facility linked to medical colleges and institutions to provide safe outpatients services in India during the COVID-19 pandemic	Observational study	**Functional:** Chemically disinfecting the facilities (80% of the facilities implementing the disinfection procedures either daily or on alternative days). Providing PPE to the physician (PPE suites available=27.4%, N95 mask available=50.9% and surgical mask available=39.3%). Training to safely manage patients with COVID-19 were provided in 78.4% of the facilities.
Gupta *et al*^42^	India	This article details measures taken by the Government of India in preparation and response to the COVID-19 pandemic	Review	**Clinical:** Strictly following infection control measures. Streamlining screening, sample collection, diagnostic and treatment protocol. S**ervice:** Categorisation of international travellers based on their COVID-19 exposure and symptoms. Implementing strict quarantine procedure for international visitors, suspected and confirmed cases. Reducing elective care provision in the hospitals. **Functional:** Provisioning infection control modalities in the healthcare facilities. Using online (e-learning) platform for training of healthcare workers. **Organisational:** Developing coordination among institutions and stakeholders (such as National Centre for Disease Control, Ministry of Health and Family Welfare, State Public Health Departments, Virus Research and Diagnostic Laboratories (VRDLs), Indian Council of Medical Research (ICMR) - National Institute of Virology).
Gupta *et al*^43^	India	The article describes the contribution of a countrywide network of VRDLs in India for scaling up testing capacity for SARS-CoV-2	Commentary or editorial	**Clinical:** The ICMR, National Institute of Virology (NIV), and Department of Health Research (DHR) coordinated with 106 VRDLs to harmonise the SOP of sample collection, shipment and reporting procedures. **Service:** Early identification and activation of VRDLs in the cities with international airports to perform real-time PCR assays Assigning specific VRDLs as sample collection site vs testing laboratories to restructure the COVID-19 testing strategy **Functional:** Adequate provision of the logistics (reagents, primers and controls) to the VRDLs from NIV by situational analysis of the inventory. **Organisational:** All public health agencies, including the Integrated Disease Surveillance Programme (IDSP), established an effective channel of communication with VRDLs at the state and regional level.
Ha *et al*^37^	Vietnam	This article highlights specific measures adopted in Vietnam for the prevention and control of COVID-19	Review	**Clinical:** Issuing Vietnamese context-specific clinical guideline for COVID-19 management. **Service:** Setting up the centre for management of clinical support specifically for patients with COVID-19. Engaging frontline health workers to provide health education, contact tracing and set-up local/home isolation facilities. **Functional:** Ensuring the provision of medical and PPE in the healthcare facilities. **Organisational:** Establishment of a Taskforce Group on COVID-19 prevention and control by including personnel from ministries, other government committees and media. Activation of Emergency Public Health Operations Centre within the General Department of Preventive Medicine to coordinate with provincial Center for Diseases Control (CDCs).
Iyengar *et al*^47^	India	This article explores the application of smartphone technology for COVID-19 surveillance and care provision	Review	**Functional:** Development of a COVID-19 tracking application, Aarogya Setu (‘Health Bridge’) for smartphone by the National Informatics Centre. Real-time triangulation of smartphone location information collected by Aarogya Setu with national COVID-19 database build by the Government of India.
Kaplan *et al*^38^	Bolivia	This article elaborates the field experience of development and implementation of COVID-19 prevention plan among Tsimane forager-horticulturalists in Bolivia	Protocol of intervention	**Service:** Organisation of community meetings to encourage the community to participate in the pandemic response. Empowering the community to regulate integration with outsiders, establish case reporting procedures and implementing isolation procedures. Organising close to community curative care delivery structure for COVID-19 and non-Covid-19 cases so that hospitalisation can be reduced to prevent cross-infection. **Functional:** Translating English educational material into Tsimane language. Ensuring an adequate supply of PPE and provision of training. Linking of clinical data with GIS data to map community spread and aid in contact tracing. **Organisational:** Putting the tribal leaders in the front and centre in the pandemic response while coordinating with other stakeholders such as regional government and public health authorities.
Lal *et al*^50^	India	This article reviews the operational protocol to ensure the safety of the orthopaedic patients and providers in the outpatient department during the COVID-19 pandemic	Review	**Clinical:** Conducting regular screening and testing of all healthcare providers. Strictly follow social distancing protocol and use of PPE while in the health facility, during the consultation, diagnostic procedure, physiotherapy and dispensing of the drug. Use of the Aarogya Setu application on their mobile phone to ensure social distancing and safety during an outpatient visit. **Service:** Restricting consultation for elective services and providing in-person consultation for a health issue that significantly affect the lifestyle of the patients. Classifying patients as ‘COVID-19 positive’, ‘COVID-19 suspected’ and ‘No history and symptom’ and organising consultation accordingly. Referring COVID-19 suspected patients to the designated fever clinic. **Functional:** Transitioning to digital scheduling, follow-up and payment by online portal or telephone.
Lucero-Prisno *et al*^39^	Africa n region(Algeria, Cameroon, Cote d’Ivoire, Gambia, Madagascar, Nigeria, Rwanda, Senegal, South Sudan, Uganda)	This article provides a commentary on the pandemic response effort of the African continent	Commentary or editorial	**Clinical:** Development of country-specific clinical case management protocol. **Service:** Coordination of a wide range of services across African countries, which includes the screening of incoming travellers at the point of entry, surveillance, community engagement for COVID-19 prevention, capacity building of the healthcare facilities for testing and case management. Building public awareness through an interactive COVID-19 dashboard. **Functional:** Conducting training and knowledge dissemination sessions with the Rapid Response Team (RRT). **Organisational:** Establishing the Africa Task Force for Novel Coronavirus by the Africa CDCs, in collaboration with the WHO. Formation of Emergency Operations Centres and RRT for cross-country collaboration.
Meghana *et al*^51^	India	This article explores the engagement of 24 pharmacy professionals (PPs) across seven states of India on Emergency Preparedness & Response of COVID-19 pandemic	Observational study	**Service:** PPs reported that they routinely screened patients for fever and cough PPs often provides telephone consultations to patients and disseminated information regarding mask use and hand washing **Functional:** Ministry of Health and Family Welfare instructed the Pharmacy Council of India to enlist pharmacists and train them as a part of the COVID-19 response (such as supply chain, inventory management, infection control and rational use of the drug).
Meghwal *et al*^52^	India	This article elaborates the field experience of COVID-19 cluster containment strategies in a healthcare facility by Central and the State RRTs at Bhilwara, Rajasthan, India	Observational study	**Service:** Implementing a door-to-door screening procedure of influenza-like illness in the district by the Mobile Health Teams. Scheduling rotational service for the physicians. **Functional:** Implementation of disinfection procedure in the health facilities and development of buffer zones. Contact tracing and implementation of isolation procedure of the discharged patients. Using the Rajasthan Social Media Platform application on smartphone devices to sure the home quarantine measures of the suspected cases. Training of all the medical, paramedical, administrative staff for implementation of containment guidelines. **Organisational:** Formation of a multidisciplinary RRT which includes experts from several departments of a state medical college, IDSP District Epidemiologist and Surveillance Medical Officer of National Polio Surveillance Programme WHO India.
Piryani *et al*^44^	Nepal	This article summarises Nepal’s response before and after WHO declared COVID-19 as a pandemic	Commentary or editorial	**Clinical:** Development of Nepal’s treatment protocol for COVID-19 sample collection, transportation and case management based on by UN Health Agency’s recommendation. Drafting and implementing the ‘Quarantine Procedure for Nepali Students repatriating from China’. **Service:** Dedicating specific space and isolation facilities to treat COVID-19 as early as January 2020. Implementing screening procedure at the Tribhuvan International Airport and ensuring safe transport of suspected cases to designated hospitals—delivering COVID-19 specific information by a free call centre. **Functional:** Building the COVID-19 diagnostic capacity of National Public Health Laboratory on 27 January 2020, following up by initiation of testing at the Provincial Public Health Laboratories from April 2020. Ensuring adequate PPE for healthcare facility and testing laboratories. **Organisational:** Formation of a high-level technical team for the pandemic response, which includes Department of Health Services, Ministry of Health and Population, Ministry of Social Development, Health Emergency Operation Centre and Provincial Health Emergency Operation Centre.
Rastogi *et al*^48^	India	This article advocates the integration of Ayurvedic therapy with Allopathic medicine to ensure effective pandemic management	Commentary or editorial	**Clinical:** Advocating Ayurvedic intervention and healthy lifestyle as prophylaxis. Recommending specific Ayurvedic treatment for COVID-19 infected patients considering their broad-spectrum antivirals properties.
Rastogi *et al*^49^	India	This article explores the opportunity of WhatsApp facilitated video Ayurveda consultation during the COVID-19 pandemic	Commentary or editorial	**Service:** Conducting online consultation as an alternative to outpatient care service. **Functional:** Assignment of an online consultation coordination team to coordinate the calls, record keeping and explaining the components of advice.
Shinde *et al*^53^	India	This article reviews the triage guideline for the surgical procedure for cancer using COVID-19 pandemic	Review	**Clinical:** Clinical decision of conducting surgery or delaying the procedure should be based on prognosis and patient’s condition—screening and diagnostic test. Surgical protocol and guidelines need to accommodate additional infection control measures, such as conducting the surgery in the operative room with negative pressure, taking extra precautions for anaesthesia-related procedures, thoracic and health-neck surgery.
Zgueb *et al*^36^	Tunisia	This article describes the development and implementation of novel psychological crisis intervention in response to the COVID-19 pandemic in Tunisia	Protocol of intervention	**Clinical:** Implementing a well-defined triage algorithm to assess any psychological crisis provide a correct referral to healthcare providers. **Service:** Providing psychological counselling via a call-centre based helpline. **Functional:** Training of volunteer students on the call centre platform and method of communication during the counselling process. **Organisational:** Coordination between the Strategic Health Operations Centre (Shoc room) of the Ministry of Health, the psychological support unit (CAP) and the national telephone operator during the development of the intervention strategy. Collaboration among the Shoc room, the CAP, Tunisian Medical Student’s Association (Associa-Med) and the Tunisian Red Crescent to build a pool of psychological counsellor.

18 studies were included in the scoping review, which met the inclusion criteria, Ayurveda is one of the traditional/complementary medicine systems practiced in India.

GIS, Global Positioning System.

When implemented at the alert phase, organisational integration emerged as a cardinal feature of the IHSD system.^36–44^ While we have observed collaboration between local, state and federal institutions for screening, isolation and case management,^36–38 41–44 51^ cross-country collaboration and partnership with international development organisations were also evident as organisational integration.^39 40 52^ Among the included studies in this review, the most common examples of functional integration—coordination between clinical and non-clinical functions—involved knowledge management and training of healthcare providers,^36 38–41 43 45 46 51 52^ maintain the inventory and supply chain of personal protective equipment, clinical equipment and medication,^37 38 41 42 44 46^ infection control of the healthcare facilities^43 46 52^ and mobilising community-based contact-tracing of recently discharged patients.^52^ We have also observed a unique archetype of functional integration where Global Positioning System and smartphones were used for contact tracing and case surveillance of COVID-19,^38 47 52^ and digital health technologies were used for teleconsultation and follow-up of routine cases to ensure social distancing measures.^49 50^

Beyond the conventional features of service integration—coordination of prevention and treatment for COVID-19 within and/or across facilities or through a team of multidisciplinary provider team^37–45 48 50 52^—some of the unique examples of service integration involved incorporating allied healthcare providers such as AYUSH (Ayurveda, Yoga and Naturopathy, Unani, Siddha and Homoeopathy—the six types of traditional or complementary medicine systems practiced in India) and pharmacy professionals (PPs) in COVID-19 response^49 51^ and organising psychological counselling helpline.^36^ Finally, as part of clinical integration, 11 studies advocated developing and implementing COVID-19 specific guidelines to ensure the coherence of rules and policies at various health systems levels.^36 37 39 41–45 48 50 53^

Shifting the perspective from the operational features of IHSD to country-level results has provided further insights into how integration approaches were adopted in various regions. Several countries from the African region (Algeria, Cameroon, Cote d’Ivoire, Gambia, Madagascar, Nigeria, Rwanda, Senegal, Sudan, South Sudan, Tunisia, Uganda) demonstrated a robust IHSD system.^36 39 40^ These integrations involved all the components of the health systems building blocks (except healthcare financing), including service delivery through community engagements for behavioural change, surveillance and monitoring programmes, leveraging technology to support information dissemination and ensuring governance through active involvement of the respective health departments. We also observed an ecosystem of partnership among different entities, such as communities and health facility teams, interdepartmental working groups, the Africa Task Force for Novel Coronavirus and the WHO.

In the context of India, the majority of IHSD cases were during the pandemic phase, except two that were observed for the alert phase.^41 43^ Most studies refer to integration mechanisms that correspond to only two or three building blocks of the health systems. Only two studies reported activities related to COVID-19 response, encompassing all the building blocks (except healthcare financing).^42 52^ Notably, we have found that the health workforce was integrated through the formation of RRTs of specialists from public health, epidemiology, respiratory medicine, paediatrics, general medicine, microbiology and otorhinolaryngology.^52^ Besides, the health systems governance structure was integrated through the coordination between the Indian Council of Medical Research and the WHO to ensure effective delivery of services,^42 43^ and health information infrastructure was integrated with service delivery systems by forming mobile health teams to ensure data monitoring and surveillance activities.^52^

Another country in the Asian region, Nepal, implemented IHSD during the alert phase and demonstrated a normative mechanism of integration.^44^ In their study, Piryani *et al*^44^ found that Nepal’s integrated response to the COVID-19 included all typologies of integration. Their study highlighted integration between service provision and technology to enable surveillance activities and inter-organisational coordination to ensure strong governance and continuity of routine service delivery.^44^ L-LMICs from the East Mediterranean region (Egypt, Morocco, Sudan and Tunisia) and South America (Bolivia) adopted a systematic approach for integration.^38 40^ Their response to COVID-19 involved three building blocks of health systems in IHSD implementation, with service delivery and governance as a common component to both. The countries from the East Mediterranean region heavily focused their effort on the alert phase. In this region, several L-LMICs (such as Egypt, Morocco, Sudan and Tunisia) coordinated with upper-middle-income (Iraq and Jordan) and high-income (Saudi Arabia) economies through the Eastern Mediterranean Public Health Network, and the Field Epidemiology Training Programmes. This multi-country coordinated effort supported a unique IHSD system to enable screening and surveillance activities, exchange information among Public Health Emergency Management Centres (PHEMC), and harmonise protocols, case definitions and public messaging strategies in the East Mediterranean region countries.

### Opportunities, challenges and recommendations to implement the IHSD system during COVID-19

Based on the review of the selected studies, we have summarised the opportunities and challenges for implementing the IHSD system in the L-LMICs during COVID-19 in table 4. We have also organised some critical recommendations that emerged from the evidence while conducting the review process.

**Table 4:** Summary of opportunities and challenges identified for implementation of the integrated health service delivery system and prospective recommendations

Phases	Integrated health service delivery implementation during COVID-19		Recommendations	
	Opportunities	Challenges	COVID-19 specific	Routine health system specific
Alert phase	Change in community behaviour driven by transparency in information and clear communication through official and social media platforms. Stewardship of the central government and decentralisation of decision-making capacity to the local authorities. Existing laboratory networks. Established telecommunication infrastructure with a high internet penetration rate.	Challenges related to inventory control of personal protective equipment (PPE) and medications. A limited supply of medical equipment such as ventilators and PPE. COVID-19 related rumours and fake news.	Strengthening of coordination between various healthcare bodies at both local, national and global level. Updating the ‘Pandemic Playbook’ with the testing, training and quarantining strategies for better disease management.	Establishing integrated platforms such as testing laboratories and electronic medical record system within routine health infrastructure, which can improve utilisation during public health emergencies.
Pandemic phase	Coordination between government ministries, public health institutions and national and international regulatory agencies. Intersectoral collaboration between government, private sector, media and armed forces. Synergies between various cadres within the health systems such as community health workers and primary care providers. Large scale application of digital health technologies such as teleconsultation, scheduling, payment portal and smartphone application for contact tracing.	A paucity of trained public health professionals, especially in epidemiology and outbreak investigation. Fragmented service delivery structure with poorly managed health information system. High burden of malnutrition, malaria, HIV/AIDS and tuberculosis which already overwhelm the health systems. Unprepared international travel infrastructures such as airports and land borders. Technological limitations related to smartphones of the end-user such as internet connectivity and availability of the required application. Privacy and data ownership issues.	Empowering communities by engaging them in disease outbreak prevention and containment strategies. Training and engaging the informal service providers such as AYUSH and community-based pharmacy professionals for pandemic prevention and response. Expansion of digital health technologies for contact tracing, inventory, and supply chain management for medication, equipment and vaccines. Ethical use of data and patient information.	Developing service delivery infrastructure using digital health technologies for prevention, treatment and follow-up of non-communicable diseases and mental health. Expanding inventory and strengthening of the supply chain to enable timely availability of medication and equipment.
Inter-pandemic	Well established network of primary health centres that ensured proper patient-centred care.	Weak public health infrastructure that can learn and adapt using previous experience. Potential delays in delivering care to other essential services (such as maternal and child health, non-communicable diseases and elective surgical procedures) due to the dispersion of human resource and physical infrastructure.	Developing robust disease surveillance and reporting mechanism. Building trust of the population in the health system. Developing a health workforce with an appropriate skill-mix that includes specialist, clinical and para-clinical workers, frontline health workers and trained informal service providers.	Building resilience of the routine health systems by increasing investment in primary healthcare and integrated care system infrastructure.

They are the six types of traditional/complementary medicine systems practiced in India; Ayurveda is one of the traditional/complementary medicine systems practiced in India.

AYUSH, Ayurveda, Yoga and Naturopathy, Unani, Siddha, Homeopathy.

In the alert and pandemic phase, existing robust health system governance structures appear to be the essential component of implementing an IHSD system in responding to the COVID-19 pandemic. Strong stewardship of the central government and confidence in the local institutions and governing bodies to take appropriate measures by understanding the context appears to be the critical factor in several studies.^37 38 42 44 52^ This type of decentralisation of the decision-making power and information needs to flow from the health systems structure down to the community level to effectively engage everyone in the pandemic preparation and response effort.^38 52^

Simultaneously, upstreaming of multisectoral collaboration within the country, and among regional and international development partners can be a vital source of sharing the most updated knowledge and resources related to COVID-19.^39–41 43^ On the other hand, poorly resourced health system with weak service delivery structure,^39 46 52 53^ fragmented supply chain,^37–39 43 46 51^ low diagnostic capacity^39 43 44^ and insufficient health workforce^46 51^ create bottlenecks to implement a well-coordinated IHSD system in L-LMICs. The key recommendation that emerged from the evidence while conducting the review process is discussed in the next section.

## Discussion

This review aimed to explore the published evidence of the IHSD system implemented during the COVID-19 pandemic to further our understanding of the structures, mechanisms and features of integrated care models in L-LMICs. We have identified 18 articles that met our inclusion and exclusion criteria and explained the reported integrated service delivery structure as part of pandemic preparedness, response and recovery.

Most of the articles focused on the pandemic phase, with some providing perspectives on the pandemic continuum’s alert phase. None of the included articles used the term ‘Integrated Health Service Delivery’ explicitly in their papers, although the authors identified aspects of integration and categorised the structure, mechanism and typologies of integration. This could indicate that the definition and nomenclature of integration adopted to synthesise the evidence in the scoping review apply to L-LMIC health systems, but the specific terminologies are not widely used in the articles. Three-fourth of the studies implemented IHSD systems that crosscut multiple typologies of the integrated model. While implementing the IHSD model, all articles reported integrating more than one health system building block for service provision, and none of them reported integrating health financing strategy as part of their IHSD approach. Health financing, as compared with other health systems building blocks, was also the least-integrated building block in a 2019 review on integrated care systems.^34^ This points to a possible evidence gap warranting further exploration.

The majority of the study systemically implemented the IHSD systems, with almost all the studies (17/18) included some type of horizontal integration, while less than half (8/18) provided examples of vertical integration. This raises some critical questions, such as—are horizontal approaches easier, or are they better suited to any healthcare emergencies, or are they more in line with pre-existing efforts at integration? While all these are important queries, the scope of this review was not designed to answer these questions, nor the articles included in this review elaborated on the result of the adopted IHSD systems in detail.

Fragmentation of health systems remains a global challenge. During the COVID-19 pandemic, the lack of integration within service delivery mechanisms became a critical factor when countries of all income levels are trying to meet the dual goal of pandemic management and routine service delivery.^54^ In L-LMICs specifically, historically verticalised and disease-oriented approaches have created additional fragmentation, which may have posed further challenges for COVID-19 pandemic preparedness and response.^30 54 55^ We have found evidence of a range of opportunities in the L-LMICs towards introducing IHSD innovations in response to the COVID-19 pandemic. The importance of existing primary healthcare and public health infrastructure was emphasised in several studies,^37 43^ and existing networks/infrastructure was identified as an enabler to integration. For example, the existing countrywide network of Virus Research and Diagnostic Laboratories in India was pivotal for scaling up testing capacity for SARS-CoV-2 by coordinating with other public health agencies at the state and national level.^43^ Similarly, in Vietnam, activation of the existing Emergency Public health Operation Centres ensured an effective integration with the Centers for Disease Control and Prevention and Department of Preventive medicine in health workers and medical supplies management.^37^ Conversely, poor existing infrastructure, weak supply chains and human resource gaps were highlighted as barriers to integration. Whether this indicates an actual pattern of pandemic response in different countries or is merely a representation of differential access to or decision to publish emerging experiences could be an area for further inquiry.

### Strengths and limitations

This review has synthesised a rapidly changing evidence based on IHSD in L-LMICs during phases during the COVID-19 pandemic. To our knowledge, this is the first review to precisely apply the definition of IHSD for COVID-19 response in the settings of L-LMICs. Much of the existing evidence on IHSD during health emergencies is conceptual in nature. This includes recommendations to strengthen national health systems vis a vis the International Health Regulations,^56 57^ emphasising an integrated approach to resilient health systems,^58^ and improving overall systems coordination.^14^ Specific evidence on IHSDs from previous health emergencies also remains sparse, possibly due to the ambiguity of conceptualising IHSD in the past. After many of the world’s most recent pandemics (eg, West Africa Ebola, MERS, SARS and H1N1), there was a rapid expansion of IHSD evidence occurred around 2015. However, there was a lack of conceptual clarity and a common definition of health service integration,^59–61^ making it particularly challenging to identify integration evidence from past pandemics, even though integration approaches could have been used. Our review contributes to this body of knowledge by synthesising the evidence of IHSD during COVID-19, which will be immensely valuable for any future pandemic response.

We also recognise that the challenges of health systems fragmentation are not specific to L-LMIC health systems^17^; however, the unique nature of IHSD reforms in L-LMICs compared with upper-middle-income and high-income settings require detailed exploration as to whether or not these approaches are being applied during COVID-19. Given the potential promise of IHSD in strengthening health systems’ resilience during health emergencies,^62 63^ an early view into IHSD approaches—or lack thereof—in L-LMICs was warranted. With a systematic approach for identifying evidence, selecting the study and analysing data, we have successfully answered our postulated research questions.

Among the eligible articles, 12 out of 18 were from India, representing an increasing focus on IHSD in the Indian health system. This may result from a higher prevalence of COVID-19 in India and a greater concentration of research institutions rapidly publishing insights from the Indian response. Besides, we have specified the inclusion criteria only for publications in English, which may have resulted in less evidence from non-Anglophone L-LMIC countries. However, due to the limited capacity of our research team, expanding the inclusion criteria to other languages (such as French and Portuguese) was not possible. The relatively sparse literature may also not represent the actual presence of IHSD approaches being used in the routine health service delivery system in L-LMICs. A significant portion of health system experiences and innovations are never documented in the peer-reviewed literature.^64^ Thus, additional research and analysis of grey literature can help to contribute additional evidence on the IHSD system in pandemic response.

Finally, the pandemic’s trajectory and a predetermined focus on L-LMICs may have limited the total number of articles identified during the early phase of the COVID-19 pandemic, as we have explored the published evidence between 1 December 2019 and 12 June 2020. We acknowledge that with the evolution of COVID-19 over the last year, new studies and evidence on the later part of the pandemic are becoming available. Thus, we are encouraging future reviews to synthesise the evidence of IHSD on the later phases of the pandemic, taking this study as a source of baseline evidence.

### Policy recommendations

Although the review did not highlight any specific patterns or characteristics of IHSD appearing in the COVID-19 literature from L-LMICs, it did indicate a range of operational approaches deployed in the early days of pandemic preparedness and response. As part of synthesising the evidence on IHSD systems, we have also identified some emerging recommendations for L-LMICs, which are critical to sustain the integrity and further build the health system’s resilience (table 4).

Specific to COVID-19 or any future pandemic, it is necessary to strengthen intersectoral coordination via organisational integration—including the private sector, laboratories and non-biomedical systems such as Ayurveda (one of the traditional medicine systems practiced in India)—while integrating the levels and building blocks of the health system. Other than supporting broader governance structure for screening, isolation and curative care provision at the health systems-level, organisational integration seems to have also played an essential role in overcoming health workforce gaps, mobilising rapid response teams, enriching technical inputs, establishing necessary infrastructures such as isolation units, quarantine centres, strengthening collaboration between surveillance units and viral research labs. Organisational integration possibly is a vital strategy to augment pandemic response in the context of L-LMICs that otherwise face health systems deficits and need additional resources from allied sectors.

Our result suggested that while implementing the IHSD system, some healthcare facilities reduced the provision of elective procedures.^43 45^ However, a similar strategy cannot be implemented for some routine service delivery systems such as obstetrics care,^65^ immunisation of children^66^ and cardiovascular emergencies.^67^ Thus, the integrated care delivery application during pandemic also needs to ensure the undisrupted provision of these critical routine care services. Alternative service delivery mechanisms such as community-based care, task-shifting using community pharmacists and volunteers for contact tracing and counselling functions, and use digital health technologies for prevention, treatment and follow-up of non-communicable diseases and mental health can spur innovations as a part of the IHSD models in L-LMICs. Finally, governments in L-LMICs need to ensure the ethical use of data and patient information,^68^ develop a transparent communication strategy to convey scientific evidence and empower the communities to be active agents for COVID-19 prevention, surveillance and containment strategies.^69^

The policy recommendations drawn in this review emerged from the analysis of the selected 18 studies representing a smaller number of L-LMICs. While we acknowledge the limited generalisability of the recommendations, they certainly are forward-looking strategies that are potential value additions to the limited pool of evidence for implementation of IHSD during COVID-19. It is essential that we refer to them as solid starting points to advocate the IHSD system and build the necessary evidence base to inform policies that can be further modified based on the country’s context, demographics and healthcare needs. Moreover, although the findings and policy recommendations were identified from COVID-19 experiences, we argue that they are not limited to the pandemic response. Barriers and facilitators to integration represent challenges for health systems strengthening more broadly,^70^ while policy recommendations to strengthen coordination, empowering communities, building trust and developing the right skills-mix for the health workforce can be equally applied to non-pandemic times.^58^ The recommendations from this study are all reflective of adaptive and resilience approaches, mirroring broader recommendations for health systems strengthening and resilience in the literature.^71^

## Conclusion

The COVID-19 pandemic was a significant shock to the health systems of L-LMICs,^5 72^ and an integrated model of health service delivery can assist the care provision of COVID-19 related illness and support the currently overwhelmed routine health service delivery structure.^25 26 73^ Using a robust—yet flexible—methodology of a scoping review, this study was able to systematically organise and report the use of an integrated care system during COVID-19, which to date was not available. We believe the evidence of IHSD presented in this review has emerged organically in response to the COVID-19 emergency that is often not documented in the literature. The results demonstrated the crux of the issue with the potential of organisational innovation capability of the health systems in the L-LMICs despite the fragmented structure and dearth of resources. However, the lack of published evidence on IHSD from L-LMICs indicates a significant gap in the original research. We hope the result of our synthesis will encourage more primary research on the integrated care system. Furthermore, we recommend future reviews to revisit the emerging evidence base on IHSD at the later phases of the COVID-19 response and recovery in L-LMICs and beyond to explore how the nascent approaches highlighted here evolve over time.

## Data Availability

Data sharing not applicable as no datasets generated and/or analysed for this study. No additional data are available. This study is developed from publicly available secondary data. The scoping review is registered on OSF.io with the Registration DOI 10.17605/OSF.IO/KY9PX (osf.io/yk7gu).
