# Supplementary material for: Integrated health service delivery during COVID-19: a scoping review of published evidence from low-income and lower-middle-income countries
Source: BMJ Glob Health. 2021 Jun 16;6(6):e005667. doi: 10.1136/bmjgh-2021-005667 (PMC8210663; doi:10.1136/bmjgh-2021-005667)
Supplement: Supplementary data [file bmjgh-2021-005667supp002.pdf]

# Integrated health service delivery during COVID-19: a scoping review of published evidence from low- and lower-middle income countries

## ***Supplemental Materials***

**Supplement 01: PubMed literature search script for the scoping review**

| Search Theme | Search Script for PubMed |
|---|---|
| Integrated care | (Delivery of Health Care, Integrated[mesh] OR Integrat*[tw] OR Integrat* Care[tw] OR Integrat* Health Care[tw] OR Integrat* Healthcare[tw] OR Integrat* Health Care System*[tw] OR Integrat* Healthcare System*[tw] OR Integrat* Care Model*[tw] OR Integrat* Delivery System*[tw] OR Integrat* Service Delivery[tw] OR Integrat* Service Delivery System*[tw] OR Integrat* Health Service*[tw] OR Integrat* Health Service* Delivery[tw] OR Integrat* Health Care Polic*[tw] OR Integrat* Healthcare Polic*[tw] OR Integrat* Health Care Organization*[tw] OR Integrat* Healthcare Organization*[tw] OR Integrat* model* of health care[tw] OR Integrat* model* of healthcare[tw] OR Health System* Integrat*[tw] OR Integrat* of Health Care System*[tw] OR Integrat* of Healthcare System*[tw] OR Integrat* of Health System*[tw] OR Integrat* of Health System*[tw] OR Service* integrat*[tw] OR System* Integrat*[tw] OR Continuity of Patient Care*[mesh] OR Healthcare Continuum[tw] OR Health Care Continuum[tw] OR Care Continuum[tw] OR Continuum of Care[tw] OR Continuum of Healthcare[tw] OR Continuum of Health Care[tw] OR Case Management*[mesh] OR Care, Patient-Centered[mesh] OR Patient Centered Care[mesh] OR Patient-Centered Care*[mesh] OR Patient-Focused Care[mesh] OR Care, Patient-Focused[mesh] OR Patient Focused Care[mesh] OR Coordinat*[tw] OR Coordinat* Care[tw] OR Coordinat* Health Care[tw] OR Coordinat* Healthcare[tw] OR Seamless Care[tw] OR Comprehensive Health Care[tw] OR Comprehensive Healthcare[tw] OR Collaborat*[tw] OR Collaboration between[tw] OR Interface*[tw] OR Case Manage*[tw] OR Case-management[tw] OR Case Management[tw] OR Patient-Centered Care*[tw] OR Patient Centered Care*[tw] OR Patient-Focused Care*[tw] OR Patient Focused Care*[tw] OR People-centred Care*[tw] OR People Centred Care*[tw] OR People-centred health system*[tw] OR People Centred health system*[tw] OR People-centered Care*[tw] OR People Centered Care*[tw] OR People-centered health system*[tw] OR People Centered health system*[tw]) AND |
| Pandemic Preparedness | ((Pandemics[MeSH] OR Pandemic*[all] OR Epidemic[MeSH] or Epidemic*[all] OR Disease Outbreaks[MeSH] OR “disease outbreaks”[all] OR “disease outbreak”[all] OR (“disease”[all] AND (“outbreaks”[all] OR “outbreak”[all]))) AND (Preparedness, Emergency[Mesh] OR Emergency Preparedness[Mesh] OR “Emergency Preparedness”[all] OR Planning, Disaster[Mesh] OR “Disaster Relief Planning”[all] OR “Disaster Relief”[all] OR Public Health Surveillance[Mesh] OR "Public Health Surveillance"[all] OR Surveillance[all] OR “Pandemic preparedness”[all] OR “pandemic planning and response”[all] OR preparedness[all] OR response[all] OR planning*[all] OR management[all] OR prevention[all] OR “humanitarian crises”[all])) AND |
| COVID-19 | ("2019 novel coronavirus disease"[tw] OR "COVID19"[tw] OR "COVID-19 pandemic"[tw] OR "SARS-CoV-2"[tw] OR "SARS-CoV-2 infection"[tw] OR "COVID19 virus"[tw] OR "COVID-19 virus"[tw] OR "COVID-19 virus disease"[tw] OR "COVID-19 virus infection"[tw] OR "COVID-19”[tw] OR “COVID19”[tw] OR "2019 novel coronavirus"[tw] OR "2019 novel coronavirus infection"[tw] OR “2019-nCoV infection"[tw] OR "coronavirus disease 2019 "[tw] OR "coronavirus disease-19"[tw] OR "2019-nCoV"[tw] OR "2019-nCoV disease" OR "Wuhan coronavirus"[tw] OR "Wuhan seafood market pneumonia virus"[tw] OR "SARS2"[tw]) AND |
| LMICs | ((Deprived Countries[tw] OR Deprived Population[tw] OR Deprived Populations[tw] OR Developing Countries[tw] OR Developing Country[tw] OR Developing Economies[tw] OR Developing Economy[tw] OR Developing Nation[tw] OR Developing Nations[tw] OR Developing Population[tw] OR Developing Populations[tw] OR Developing World[tw] OR LAMI Countries[tw] OR LAMI Country[tw] OR Less Developed Countries[tw] OR Less Developed Country[tw] OR Less Developed Economies [tw] OR Less Developed Nation[tw] OR Less Developed Nations[tw] OR Less Developed World[tw] OR Lesser Developed Countries[tw] OR Lesser Developed Nations[tw] OR LMIC[tw] OR LMICS[tw] OR Low GDP[tw] OR Low GNP[tw] OR Low Gross Domestic[tw] OR Low Gross National[tw] OR Low Income Countries[tw] OR Low Income Country[tw] OR Low Income Economies [tw] OR Low Income Economy[tw] OR Low Income Nations[tw] OR Low Income Population[tw] OR Low Income Populations[tw] OR Lower GDP[tw] OR lower gross domestic[tw] OR Lower Income Countries[tw] OR Lower Income Country[tw] OR Lower Income Nations[tw] OR Lower Income Population[tw] OR Lower Income Populations[tw] OR Middle Income Countries[tw] OR Middle Income Country[tw] OR Middle Income Economies [tw] OR Middle Income Nation[tw] OR Middle Income Nations[tw] OR Middle Income Population[tw] OR Middle Income Populations[tw] OR Poor Countries[tw] OR Poor Country[tw] OR Poor Economies [tw] OR Poor Economy[tw] OR Poor Nation[tw] OR Poor Nations[tw] OR Poor Population[tw] OR Poor Populations[tw] OR poor world[tw] OR Poorer Countries[tw] OR Poorer Economies [tw] OR Poorer Economy[tw] OR Poorer Nations[tw] OR Poorer Population[tw] OR Poorer Populations[tw] OR Third World[tw] OR Transitional Countries[tw] OR Transitional Country[tw] OR Transitional Economies[tw] OR Transitional Economy[tw] OR Under Developed Countries[tw] OR Under Developed Country[tw] OR under developed nations[tw] OR Under Developed World[tw] OR Under Served Population[tw] OR Under Served Populations[tw] OR Underdeveloped Countries[tw] OR Underdeveloped Country[tw] OR underdeveloped economies[tw] OR underdeveloped nations[tw] OR underdeveloped population[tw] OR Underdeveloped World[tw] OR |

| | |
|---|---|
| | Underserved Countries[tw] OR Underserved Nations[tw] OR Underserved Population[tw] OR Underserved Populations[tw])<br>OR<br>(Afghanistan[tw] OR Angola[tw] OR Bangladesh[tw] OR Benin[tw] OR Bhutan[tw] OR Bolivia[tw] OR Burma[tw] OR “Burkina Faso”[tw] OR Burundi[tw] OR “Cabo Verde”[tw] OR “Cape verde”[tw] OR Cambodia[tw] OR Cameroon[tw] OR “Central African Republic”[tw] OR Chad[tw] OR Comoros[tw] OR Comores[tw] OR Comoro[tw] OR Congo[tw] OR “Côte d'Ivoire”[tw] OR Djibouti[tw] OR Egypt[tw] OR “El Salvador”[tw] OR Eritrea[tw] OR Ethiopia[tw] OR Gambia[tw] OR Gaza[tw] OR Ghana[tw] OR Guinea[tw] OR “Guinea Bissau”[tw] OR Haiti[tw] OR Honduras[tw] OR India[tw] OR Indonesia[tw] OR Kenya[tw] OR Kiribati[tw] OR Kyrgyz[tw] OR Kirghizia[tw] OR Kirghiz[tw] OR Kirgizstan[tw] OR Kyrgyzstan[tw] OR “Lao PDR”[tw] OR Laos[tw] OR Lesotho[tw] OR Liberia[tw] OR Madagascar[tw] OR Malawi[tw] OR Mali[tw] OR Mauritania[tw] OR Micronesia[tw] OR Moldova[tw] OR Mongolia[tw] OR Morocco[tw] OR Mozambique[tw] OR Myanmar[tw] OR Nepal[tw] OR Nicaragua[tw] OR Niger[tw] OR Nigeria [tw] OR Pakistan [tw] OR Palau[tw] OR Panama[tw] OR “Papua New Guinea”[tw] OR Philippines[tw] OR Phillippines[tw] OR Philipines[tw] OR Phillipines[tw] OR Principe[tw] OR Rwanda[tw] OR Ruanda[tw] OR “Sao Tome”[tw] OR Senegal[tw] OR “Sierra Leone”[tw] OR “Solomon Islands”[tw] OR Somalia[tw] OR “South Sudan”[tw] OR Sudan[tw] OR Swaziland[tw] OR Syria[tw] OR “Syrian Arab Republic”[tw] OR Tajikistan[tw] OR Tadzhikistan[tw] OR Tadjikistan[tw] OR Tadzhik[tw] OR Tanzania[tw] OR Timor[tw] OR Togo[tw] OR Tunisia[tw] OR Uganda[tw] OR Ukraine[tw] OR Uzbek[tw] OR Uzbekistan[tw] OR Vanuatu[tw] OR Vietnam[tw] OR “West Bank”[tw] OR Yemen[tw] OR Zambia[tw] OR Zimbabwe[tw]))<br>AND |
| Time frame | ( "2019/12/01"[PDat] : "2020/06/12"[PDat] ) |

*Note: Low-income economies are defined as Gross National Income (GNI) per capita of $1,035 or less in 2019 (N = 29)*
*Lower-middle income economies are defined as Gross National Income (GNI) per capita $1,036 and $4,045 (N = 50)*
*(https://datahelpdesk.worldbank.org/knowledgebase/articles/906519-world-bank-country-and-lending-groups, accessed 26 April 2020)*

## Supplement 02: Data extraction template

| *Data extraction themes* | **Data elements that will be extracted from each eligible article** | |
|---|---|---|
| *Study characteristics* | • Database<br>• Title<br>• Authors<br>• Year<br>• Country name(s) or geography | • Country income classification (World Bank)<br>• WHO region<br>• Study populations<br>• Study design and methodology |
| *Dimensions of Integrated Care and Pandemic Preparedness* | • Pandemic phase when IHSD implemented<br>• Typologies of integration<br>• Type(s) of service(s) integrated (if applicable)<br>• Integration mechanism (if applicable)<br>• Integration structure (if applicable)<br>• Integration level (if applicable)<br>• Rationale for integration (if applicable) | • Individual / Organization / Actor that Championed Integration<br>• Organizational and operational components of integration – Policy options used<br>• Organizational and operational components of integration – Implementation strategy used |
| *Intersection with COVID-19* | • Facilitators of integration<br>• Barriers to integration<br>• Positive effects of integration | • Negative consequences of integration<br>• Recommendations – COVID-19 specific<br>• Recommendations – Routine health system (non-COVID-19 specific) |

*Note: Low-income economies are defined as Gross National Income (GNI) per capita of $1,035 or less in 2019 (N = 29)*
*Lower-middle income economies are defined as Gross National Income (GNI) per capita $1,036 and $4,045 (N = 50)*
*(https://datahelpdesk.worldbank.org/knowledgebase/articles/906519-world-bank-country-and-lending-groups, accessed 26 April 2020)*

**Supplement 03: Data extraction sheet that was used for charting the evidence**

| | A | B | C | D | E | F | G | H | I | J | K | L | M | N | O | P | Q | R | S | T | U | V | W | X | Y |
|---|---|---|---|---|---|---|---|---|---|---|---|---|---|---|---|---|---|---|---|---|---|---|---|---|---|
| 1 | Study characteristics | | | | | | | | | Dimensions of Integrated Care and Pandemic Preparedness | | | | | | | | | | Intersection with COVID-19 | | | | | |
| 2 | Database ID | Title | Author (First Author) - Eg. Gupta, 2020 | Year | Country/ Geography Name | Country/R egion Type | WHO Region | Study Populatio n | Study Design and Methodolog y | Pandemic Phase When IHSD Implement ed | Typologies of Integration | Type of Service Integration | Integration Mechanism | Integration Structure | Integration Level | Rationale for Integration | Individual / Organization / Actor that Championed Integration | Organizational and Operational Components | | Facilitators of Integration | Barriers to Integration | 'Positive' effects of integration | Negative effects' of integration | Recommendations | |
| 3 | | | | | | | | | | | | | | | | | | Policy Options Used | Implementati on Strategy Used | | | | | COVID-19 Specific | Routine health system |
| 4 | | | | | | | | | | | | | | | | | | | | | | | | | |

**Supplement 04: List of low and lower-middle income countries**

| World Bank list of economies | | | |
|---|---|---|---|
| **Sr. No.** | **Economy** | **Region** | **Income group** |
| 1 | Afghanistan | South Asia | Low income |
| 2 | Algeria | Middle East & North Africa | Lower middle income |
| 3 | Angola | Sub-Saharan Africa | Lower middle income |
| 4 | Bangladesh | South Asia | Lower middle income |
| 5 | Benin | Sub-Saharan Africa | Lower middle income |
| 6 | Bhutan | South Asia | Lower middle income |
| 7 | Bolivia | Latin America & Caribbean | Lower middle income |
| 8 | Burkina Faso | Sub-Saharan Africa | Low income |
| 9 | Burundi | Sub-Saharan Africa | Low income |
| 10 | Cabo Verde | Sub-Saharan Africa | Lower middle income |
| 11 | Cambodia | East Asia & Pacific | Lower middle income |
| 12 | Cameroon | Sub-Saharan Africa | Lower middle income |
| 13 | Central African Republic | Sub-Saharan Africa | Low income |
| 14 | Chad | Sub-Saharan Africa | Low income |
| 15 | Comoros | Sub-Saharan Africa | Lower middle income |
| 16 | Congo, Dem. Rep. | Sub-Saharan Africa | Low income |
| 17 | Congo, Rep. | Sub-Saharan Africa | Lower middle income |
| 18 | Côte d'Ivoire | Sub-Saharan Africa | Lower middle income |
| 19 | Djibouti | Middle East & North Africa | Lower middle income |
| 20 | Egypt, Arab Rep. | Middle East & North Africa | Lower middle income |
| 21 | El Salvador | Latin America & Caribbean | Lower middle income |
| 22 | Eritrea | Sub-Saharan Africa | Low income |
| 23 | Eswatini | Sub-Saharan Africa | Lower middle income |
| 24 | Ethiopia | Sub-Saharan Africa | Low income |
| 25 | Gambia, The | Sub-Saharan Africa | Low income |
| 26 | Ghana | Sub-Saharan Africa | Lower middle income |
| 27 | Guinea | Sub-Saharan Africa | Low income |
| 28 | Guinea-Bissau | Sub-Saharan Africa | Low income |
| 29 | Haiti | Latin America & Caribbean | Low income |
| 30 | Honduras | Latin America & Caribbean | Lower middle income |

| | | | |
|---|---|---|---|
| 31 | India | South Asia | Lower middle income |
| 32 | Kenya | Sub-Saharan Africa | Lower middle income |
| 33 | Kiribati | East Asia & Pacific | Lower middle income |
| 34 | Korea, Dem. People's Rep. | East Asia & Pacific | Low income |
| 35 | Kyrgyz Republic | Europe & Central Asia | Lower middle income |
| 36 | Lao PDR | East Asia & Pacific | Lower middle income |
| 37 | Lesotho | Sub-Saharan Africa | Lower middle income |
| 38 | Liberia | Sub-Saharan Africa | Low income |
| 39 | Madagascar | Sub-Saharan Africa | Low income |
| 40 | Malawi | Sub-Saharan Africa | Low income |
| 41 | Mali | Sub-Saharan Africa | Low income |
| 42 | Mauritania | Sub-Saharan Africa | Lower middle income |
| 43 | Micronesia, Fed. Sts. | East Asia & Pacific | Lower middle income |
| 44 | Moldova | Europe & Central Asia | Lower middle income |
| 45 | Mongolia | East Asia & Pacific | Lower middle income |
| 46 | Morocco | Middle East & North Africa | Lower middle income |
| 47 | Mozambique | Sub-Saharan Africa | Low income |
| 48 | Myanmar | East Asia & Pacific | Lower middle income |
| 49 | Nepal | South Asia | Lower middle income |
| 50 | Nicaragua | Latin America & Caribbean | Lower middle income |
| 51 | Niger | Sub-Saharan Africa | Low income |
| 52 | Nigeria | Sub-Saharan Africa | Lower middle income |
| 53 | Pakistan | South Asia | Lower middle income |
| 54 | Papua New Guinea | East Asia & Pacific | Lower middle income |
| 55 | Philippines | East Asia & Pacific | Lower middle income |
| 56 | Rwanda | Sub-Saharan Africa | Low income |
| 57 | São Tomé and Principe | Sub-Saharan Africa | Lower middle income |
| 58 | Senegal | Sub-Saharan Africa | Lower middle income |
| 59 | Sierra Leone | Sub-Saharan Africa | Low income |
| 60 | Solomon Islands | East Asia & Pacific | Lower middle income |
| 61 | Somalia | Sub-Saharan Africa | Low income |
| 62 | South Sudan | Sub-Saharan Africa | Low income |
| 63 | Sri Lanka | South Asia | Lower middle income |

| | | | |
|---|---|---|---|
| 64 | Sudan | Sub-Saharan Africa | Low income |
| 65 | Syrian Arab Republic | Middle East & North Africa | Low income |
| 66 | Tajikistan | Europe & Central Asia | Low income |
| 67 | Tanzania | Sub-Saharan Africa | Lower middle income |
| 68 | Timor-Leste | East Asia & Pacific | Lower middle income |
| 69 | Togo | Sub-Saharan Africa | Low income |
| 70 | Tunisia | Middle East & North Africa | Lower middle income |
| 71 | Uganda | Sub-Saharan Africa | Low income |
| 72 | Ukraine | Europe & Central Asia | Lower middle income |
| 73 | Uzbekistan | Europe & Central Asia | Lower middle income |
| 74 | Vanuatu | East Asia & Pacific | Lower middle income |
| 75 | Vietnam | East Asia & Pacific | Lower middle income |
| 76 | West Bank and Gaza | Middle East & North Africa | Lower middle income |
| 77 | Yemen, Rep. | Middle East & North Africa | Low income |
| 78 | Zambia | Sub-Saharan Africa | Lower middle income |
| 79 | Zimbabwe | Sub-Saharan Africa | Lower middle income |
| Data source: https://datahelpdesk.worldbank.org/knowledgebase/articles/906519-world-bank-country-and-lending-groups | | | |
